# Impact of moderate-to-severe coronary calcification on 1-year clinical outcomes after IVUS-guided PCI

**DOI:** 10.3389/fcvm.2026.1720958

**Published:** 2026-02-06

**Authors:** Thanh Cong Nguyen, Vu Hoang Vu, Bao Thien Duong, Hoa Tran, Khoi Minh Le, Hung Minh Ngo, Van Hoang, Thai Quoc Nguyen, Binh Quang Truong

**Affiliations:** 1 Interventional Cardiology Department, University Medical Center Ho Chi Minh City, Ho Chi Minh City, Vietnam; 2 School of Medicine, University of Medicine and Pharmacy at Ho Chi Minh City, Ho Chi Minh City, Vietnam; 3 Cardiac Imaging Unit, University Medical Center Ho Chi Minh City, Ho Chi Minh City, Vietnam; 4 Interventional Cardiology Department, Cho Ray Hospital, Ho Chi Minh City, Vietnam; 5 Interventional Cardiology Department, Hanoi Heart Hospital, Hanoi, Vietnam; 6 Vietnam National Heart Institute, Bach Mai Hospital, Hanoi, Vietnam

**Keywords:** coronary artery calcification, coronary artery disease, intravascular ultrasound, major adverse cardiac events, percutaneous coronary intervention

## Abstract

**Background:**

Moderate-to-severe coronary artery calcification (CAC) poses major challenges during percutaneous coronary intervention (PCI) and has historically been associated with procedural failure and adverse outcomes. However, its prognostic relevance in the era of contemporary intravascular imaging–guided PCI remains uncertain. This study aimed to evaluate the impact of moderate-to-severe CAC on 1-year clinical outcomes after IVUS-guided PCI.

**Methods:**

This prospective, single-center study enrolled 914 patients who underwent IVUS-guided PCI between March and November 2023. CAC severity was classified by IVUS as none/mild or moderate-to-severe. After 1:1 propensity score matching, 576 patients (288 per group) were included. The primary endpoint was 1-year major adverse cardiac events (MACE), defined as a composite of all-cause death, myocardial infarction, and target lesion revascularization.

**Results:**

Moderate-to-severe CAC was present in 37.0% of patients. Before matching, the 1-year MACE rate was higher in patients with moderate-to-severe CAC than in those with none/mild CAC (9.5% vs. 4.2%; *p* = 0.001). In the matched cohort, this difference was attenuated and no longer statistically significant (9.4% vs. 6.3%; *p* = 0.162). Moderate-to-severe CAC was not significantly associated with MACE (HR 1.54; 95% CI 0.85–2.79; *p* = 0.157). Rates of stent thrombosis and target lesion revascularization were low and comparable.

**Conclusions:**

Under routine IVUS-guided PCI, moderate-to-severe coronary calcification was associated with 1-year clinical outcomes comparable to those of no or mild calcification after adjustment.

## 1 Introduction

Coronary artery calcification (CAC), observed in 18%–30% of patients undergoing percutaneous coronary intervention (PCI), remains one of the most formidable challenges in interventional cardiology (1–3). Moderate-to-severe CAC reduces vessel compliance, hinders device delivery, and compromises stent expansion, factors that increase the risk of procedural failure, in-stent restenosis, and major adverse cardiac events (MACE) (1, 4–6). These limitations were particularly pronounced during the era of balloon angioplasty and bare-metal stents, when inadequate plaque modification often resulted in elastic recoil, underexpansion, and poor long-term outcomes (7).

Over the past decade, PCI has evolved with the introduction of newer-generation drug-eluting stents, specialized calcium-modification devices, and high-resolution intravascular imaging (8). As the prevalence of CAC continues to rise due to aging and increasing burdens of diabetes, chronic kidney disease, and dyslipidemia, the need for optimized strategies is urgent (9). Intravascular ultrasound (IVUS) has emerged as a cornerstone in the treatment of complex coronary disease, enabling precise assessment of calcium burden and distribution, guiding lesion preparation, optimizing stent sizing and deployment, and providing post-PCI quality control (10–12). Accumulating evidence from randomized trials and meta-analyses has demonstrated that IVUS-guided PCI improves procedural and clinical outcomes, especially in complex lesions (13–15).

Nevertheless, whether moderate-to-severe CAC remains an independent predictor of adverse outcomes in the era of routine IVUS guidance and systematic lesion preparation remains unclear. To address this gap, we conducted a prospective study to evaluate the prognostic impact of IVUS-defined moderate-to-severe CAC on 1-year MACE in patients undergoing contemporary PCI.

## 2 Methods

### 2.1 Study design and population

This was a prospective, observational, single-center cohort study conducted at the University Medical Center Ho Chi Minh City, Vietnam, between March and November 2023. The study aimed to evaluate the prognostic impact of moderate-to-severe CAC on clinical outcomes in patients undergoing PCI guided by routine IVUS, systematic lesion preparation, and implantation of new-generation drug-eluting stents (DES).

**Figure 1:**
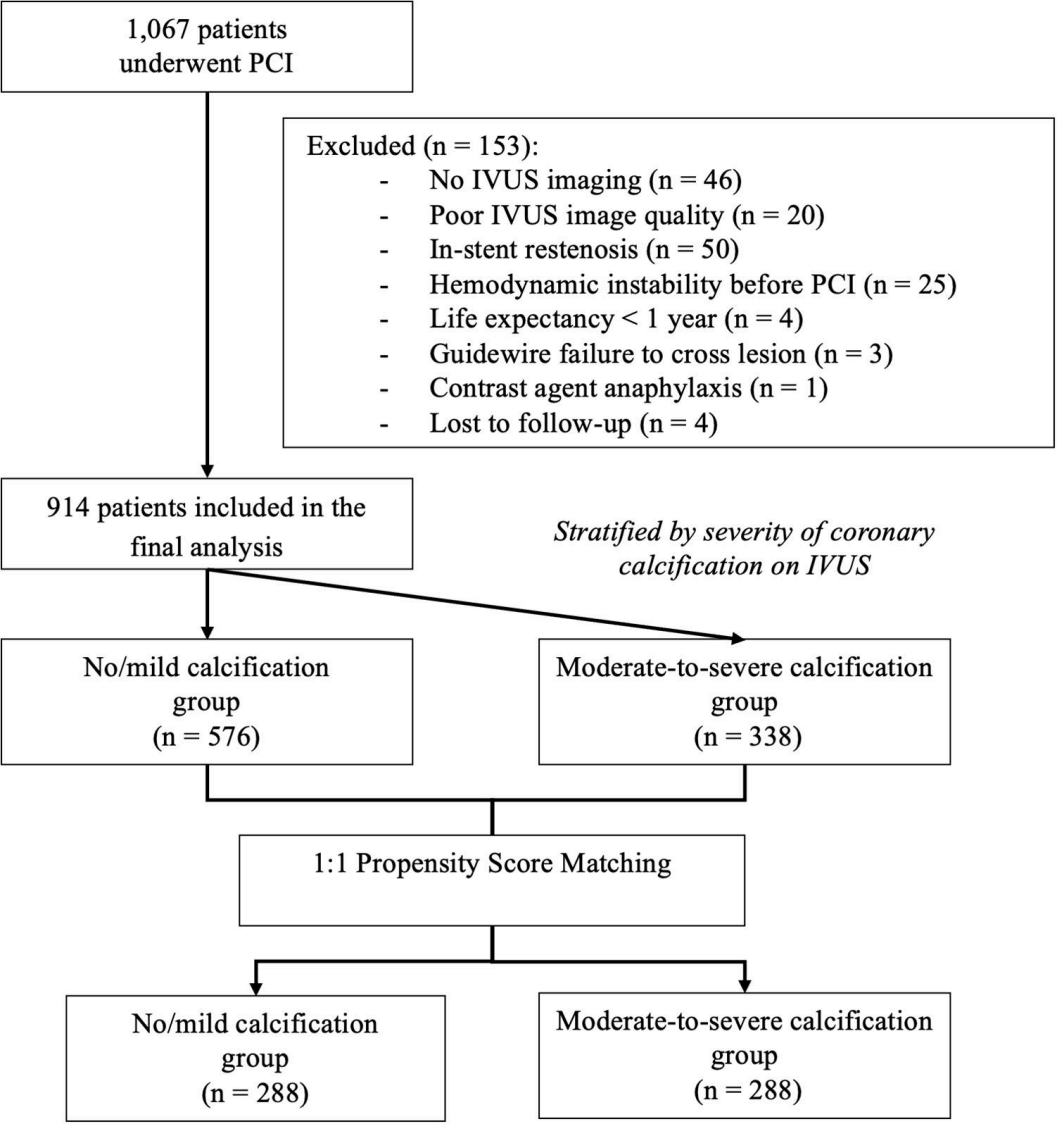
Flowchart of patient selection and IVUS-based calcification stratification. IVUS, intravascular ultrasound; PCI, percutaneous coronary intervention.

Consecutive patients were prospectively enrolled in an institutional PCI registry and participated in a structured post-PCI management program (Supplementary Method 1) (ClinicalTrials.gov Identifier: NCT06071741). Of the 1,067 patients screened, 914 met the eligibility criteria and completed 12 months of follow-up. CAC severity was assessed using IVUS, and patients were categorized into two groups: no or mild CAC (*n* = 576) and moderate-to-severe CAC (*n* = 338). To minimize confounding from baseline imbalances, propensity score matching (PSM) was performed using a 1:1 nearest-neighbor algorithm, yielding 288 matched pairs (*n* = 576). A study flow diagram is presented in Figure 1.

### 2.2 Eligibility criteria

Patients were eligible if they were ≥18 years of age, had angiographically confirmed coronary artery disease, including either chronic or acute coronary syndromes, and underwent IVUS-guided PCI according to current guidelines (16, 17). For patients with complex coronary anatomy (e.g., left main disease or multivessel involvement), the decision to proceed with PCI was made by a multidisciplinary Heart Team based on favorable anatomy and informed consent after detailed discussion with the patient or a legal representative.

Exclusion criteria included: (1) absence or poor-quality IVUS imaging precluding reliable assessment of calcification or stent optimization; (2) PCI performed for in-stent restenosis or saphenous vein graft lesions; (3) hemodynamic instability or cardiogenic shock prior to PCI; (4) contraindications to antithrombotic therapy; (5) an estimated life expectancy of <1 year; and (6) inability or unwillingness to complete 12-month follow-up. Full details are in Supplementary Method 2.

### 2.3 Calcification assessment

**Figure 2:**
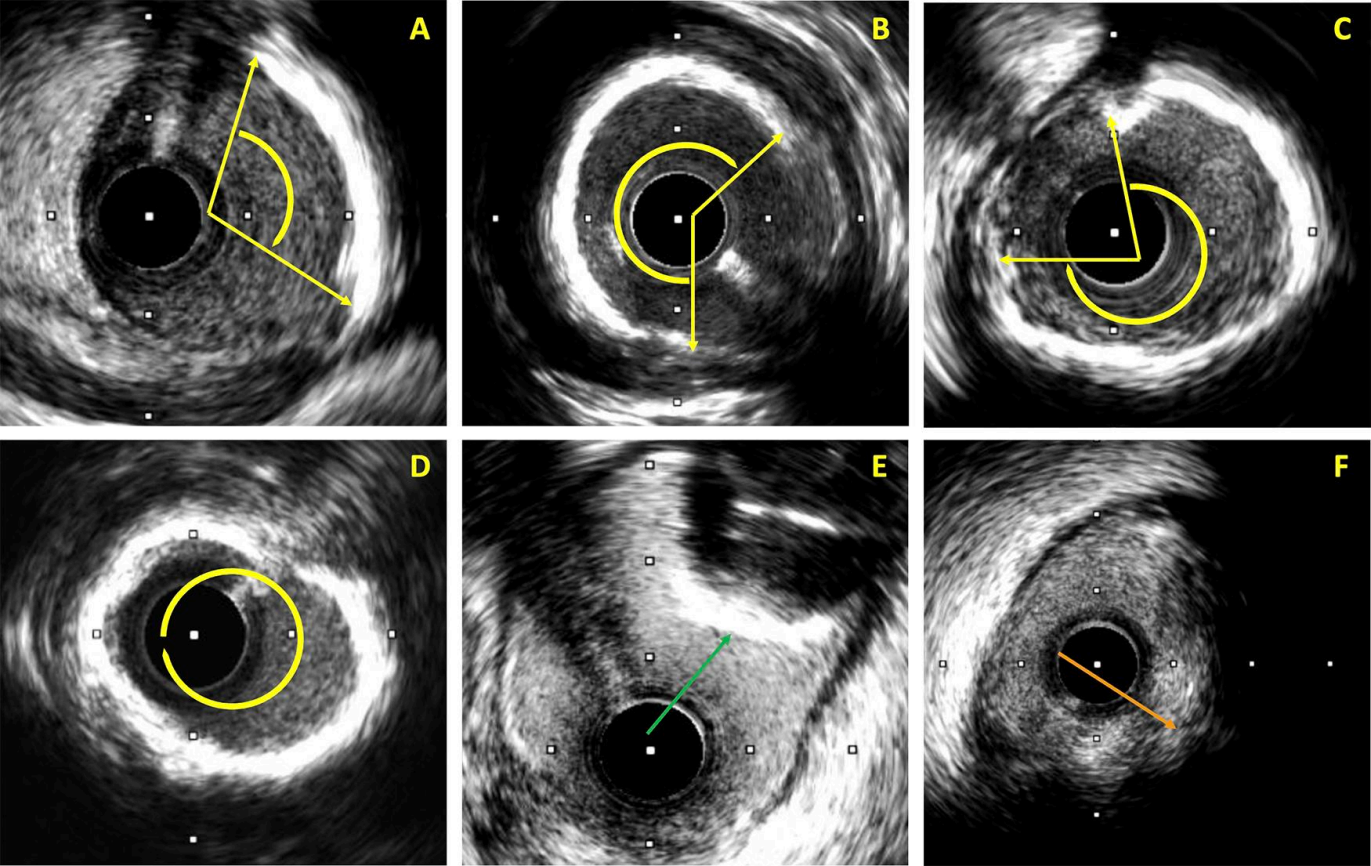
Representative intravascular ultrasound images illustrating the assessment of coronary calcification. **(A)** Mild calcification (calcium arc <180°); **(B)** moderate calcification (calcium arc 180°–270°); **(C)** severe calcification (calcium arc >270°); **(D)** circumferential (360°) ring calcification; **(E)** calcified nodule (green arrow); **(F)** hypoechoic plaque (orange arrow) with posterior acoustic shadowing not related to calcification.

Coronary calcification was assessed using IVUS, identified as hyperechoic plaque with acoustic shadowing (Figure 2). Severe calcification was defined as a calcium arc >270° extending ≥5 mm or concentric (360°) calcification on cross-sectional IVUS images. Moderate calcification was a calcium arc >180° on any frame without meeting severe criteria. Lesions below these thresholds were classified as no/mild calcification. Additional details on calcium arc quantification, distribution, calcium nodules, and lesion length are provided in the Supplementary Method 3. For patient-level classification, in patients with multiple target lesions within the same vessel or multivessel PCI during the index hospitalization, CAC severity was determined by the lesion with the highest degree of calcification. Although an independent core laboratory was not used, intra- and inter-observer reproducibility analyses performed in a randomly selected subset demonstrated high agreement for IVUS-based calcification assessment (Supplementary Result 1, Supplementary Tables 1, 2).

### 2.4 IVUS-guided PCI procedure

All procedures were performed using contemporary PCI techniques under IVUS guidance. IVUS was systematically applied throughout the procedure to characterize lesion morphology, guide calcium modification strategy, assess the adequacy of lesion preparation, optimize stent sizing and positioning, confirm post-deployment results, and detect procedural complications.

Optimal stent implantation, as assessed by IVUS, was defined as a minimum stent area (MSA) ≥5.0 mm^2^ for vessels ≥3.0 mm (excluding the left main), or ≥90% of the distal reference lumen area. For left main lesions, an MSA ≥10.0 mm^2^ was required. Major edge dissections and significant stent malapposition were not permitted. Detailed IVUS acquisition protocols and additional stent optimization criteria are provided in Supplementary Methods 4, 5.

### 2.5 Data collection and follow-up

Baseline demographic, clinical, angiographic, IVUS, and procedural characteristics were prospectively collected via standardized electronic case report forms within a dedicated PCI registry. IVUS measurements were initially acquired and documented by trained technicians and operators, then reviewed and validated by experienced interventional cardiologists. All IVUS images were archived in the institutional Picture Archiving and Communication System (PACS), and analyses were linked to clinical and procedural records via the electronic medical record (EMR) system.

All data infrastructure adhered to EuroHeart data standards, with registry implementation using the REDCap (Research Electronic Data Capture) platform. Data quality assurance included real-time validation rules, source data verification against electronic health records, and periodic audits by principal investigators and trained research staff.

Following PCI, all patients were enrolled in a structured post-PCI management program and were scheduled for standardized clinical follow-up visits at 1, 3, 6, and 12 months. Follow-up information was obtained through outpatient visits and/or structured telephone interviews, encompassing survival status, hospitalizations, recurrent symptoms, repeat revascularization, and other adverse cardiac events. When applicable, hospital discharge summaries and medical records were reviewed to confirm reported outcomes.

### 2.6 Endpoints and outcomes

The primary endpoint was 1-year MACE, comprising all-cause death, myocardial infarction, and target lesion revascularization (TLR). Myocardial infarction was classified according to the Fourth Universal Definition of Myocardial Infarction. TLR was defined as any repeat percutaneous or surgical revascularization involving the stented segment or within 5 mm proximal or distal to the stent.

Secondary endpoints included the individual components of the primary endpoint (all-cause death, myocardial infarction, and TLR), along with cardiac death and stroke. Detailed definitions of all clinical and procedural variables are provided in Supplementary Method 6.

### 2.7 Ethical considerations

The study was conducted in accordance with the Declaration of Helsinki and was approved by the institutional ethics committee of the University Medical Center, Ho Chi Minh City, Vietnam (Approval No. 108/GCN-HĐĐĐ; October 7, 2022). Written informed consent was obtained from all participants prior to enrollment.

### 2.8 Statistical analysis

Continuous variables were expressed as mean ± SD or median (IQR) and compared using the Student's *t*-test or Mann–Whitney *U* test, as appropriate. Categorical variables were presented as counts and percentages, and compared using the chi-square test or Fisher's exact test.

To reduce baseline imbalances, PSM was performed using a 1:1 nearest-neighbor algorithm with a caliper width of 0.2 SD of the logit of the propensity score. Covariates included in the propensity model were age, sex, hypertension, diabetes mellitus, chronic kidney disease, prior myocardial infarction, and presentation with acute coronary syndrome. Covariate balance after matching was assessed using standardized mean differences.

All outcome analyses were conducted in the propensity-matched cohort. Kaplan–Meier survival analysis was used to estimate cumulative event rates, and survival curves were compared using the log-rank test. Univariable Cox proportional hazards regression was performed to identify predictors of MACE, with results reported as hazard ratios (HRs) and 95% confidence intervals (CIs).

Statistical significance was set at *p* < 0.05 (two-sided). All analyses were performed using SPSS version 26.0 (IBM Corp., Armonk, NY) and R version 4.5.0 (R Foundation for Statistical Computing, Vienna, Austria).

Sample size calculation was performed using both a two-proportion comparison and a log-rank method based on anticipated 1-year event rates and hazard ratio assumptions. The minimum required sample size ranged from 727 to 876 patients. To ensure sufficient statistical power, 914 patients were enrolled (Supplementary Method 7).

## 3 Results

### 3.1 Baseline characteristics

Before PSM, patients with moderate-to-severe CAC were older, more often male, and had more cardiovascular risk factors and comorbidities than those with no or mild CAC. They showed higher rates of hypertension, prior myocardial infarction, heart failure, and atrial fibrillation. The prevalence of diabetes mellitus, chronic kidney disease, renal replacement therapy, and peripheral artery disease was also higher in this group.

After matching, baseline characteristics were well balanced between the two groups. However, patients in the moderate-to-severe CAC group continued to exhibit a higher prevalence of peripheral artery disease and renal replacement therapy. To account for the potential impact of these residual imbalances, additional sensitivity and interaction analyses were performed, as detailed in Supplementary Result 2, Supplementary Table 3.

Acute coronary syndrome was the predominant clinical presentation in both groups (88.7% in the no or mild CAC group vs. 83.7% in the moderate-to-severe CAC group), although patients with moderate-to-severe CAC had a higher proportion of stable coronary artery disease (16.3% vs. 11.3%; *p* = 0.031) and a lower incidence of ST-elevation myocardial infarction (22.8% vs. 31.1%; *p* = 0.007). These differences were no longer statistically significant after matching. Detailed clinical characteristics are provided in Table 1, and laboratory characteristics in Supplementary Table 4.

**Table 1:** Baseline clinical characteristics.

Variables	Before PSM			After PSM		
	No/mild calcification (*n* = 576)	Moderate/severe calcification (*n* = 338)	*P* value	No/mild calcification (*n* = 288)	Moderate/severe calcification (*n* = 288)	*P* value
Baseline characteristics						
Age (years)	61.3 ± 11.5	69.1 ± 9.4	<0.001	68.1 ± 9.7	67.7 ± 9.0	0.644
Male	418 (72.6)	200 (59.2)	<0.001	190 (60.1)	173 (60.1)	0.142
BMI (kg/m^2^)	23.8 ± 3.2	23.1 ± 2.9	0.001	23.1 ± 3.1	23.1 ± 3.0	0.797
Hypertension	423 (73.4)	294 (87.0)	<0.001	249 (86.5)	245 (85.1)	0.633
Prior MI	80 (13.9)	65 (19.2)	0.033	51 (17.7)	56 (19.4)	0.592
Prior PCI	56 (9.7)	44 (13.0)	0.123	28 (9.7)	39 (13.5)	0.153
Prior CABG	0 (0.0)	3 (0.9)	0.050	0 (0.0)	1 (0.3)	1.000
Heart failure	56 (9.7)	85 (25.1)	<0.001	47 (16.3)	55 (19.1)	0.383
Atrial fibrillation	15 (2.6)	19 (5.6)	0.020	12 (4.2)	11 (3.8)	0.831
Diabetes mellitus	180 (31.3)	157 (46.4)	<0.001	111 (38.5)	120 (41.7)	0.444
On insulin	40 (7.0)	39 (11.6)	0.018	25 (8.8)	28 (9.8)	0.695
Chronic kidney disease	38 (6.6)	68 (20.1)	<0.001	35 (12.2)	47 (16.3)	0.152
Renal replacement therapy	5 (0.9)	17 (5.0)	<0.001	2 (0.7)	15 (5.2)	0.002
Prior Stroke	41 (7.1)	36 (10.7)	0.063	27 (9.4)	29 (10.1)	0.778
Peripheral artery disease	21 (3.6)	50 (14.8)	<0.001	15 (5.2)	41 (14.2)	<0.001
COPD	16 (2.8)	23 (6.8)	0.004	11 (3.8)	20 (6.9)	0.097
Clinical diagnosis						
CCS	65 (11.3)	55 (16.3)	0.031	39 (13.5)	46 (16.0)	0.411
Unstable angina	162 (28.1)	86 (25.4)	0.379	85 (29.5)	79 (27.4)	0.580
NSTEMI	170 (29.5)	120 (35.5)	0.060	70 (24.3)	65 (22.6)	0.623
STEMI	179 (31.1)	77 (22.8)	0.007	94 (32.6)	98 (34.0)	0.724
ACS	511 (88.7)	283 (83.7)	0.031	249 (86.5)	242 (84.0)	0.411
Clinical presentation on admission						
Heart rate (beats/min)	80 (68.0–90.0)	81.5 (70.0–96.0)	0.612	78.0 (68.0–88.5)	79.5 (68.5–93)	0.230
Systolic blood pressure (mmHg)	130.0 (110.0–140.0)	124.5 (110.0–140.0)	0.712	130.0 (113.0–140.0)	130.0 (115.0–140.0)	0.917
Killip classification						
Killip I	532 (92.4)	287 (84.9)	<0.001	259 (89.9)	249 (86.5)	0.197
Killip II	19 (3.3)	20 (5.9)	0.059	14 (4.9)	16 (5.5)	0.708
Killip III	24 (4.2)	27 (8.0)	0.015	14 (4.9)	19 (6.6)	0.370
Killip IV	1 (0.2)	4 (1.2)	0.065	1 (0.3)	4 (1.4)	0.373
Acute heart failure	44 (7.6)	51 (15.1)	<0.001	29 (10.1)	39 (13.5)	0.197
Discharge medications						
Aspirin	560 (98.1)	330 (97.9)	0.875	275 (96.8)	282 (98.3)	0.270
Clopidogrel	242 (42.4)	167 (49.6)	0.036	145 (51.1)	134 (46.7)	0.297
Ticagrelor	329 (57.6)	170 (50.4)	0.036	139 (48.9)	153 (53.3)	0.297
Anticoagulant	21 (3.7)	23 (6.8)	0.033	17 (6.0)	14 (4.9)	0.340
Statin	571 (100.0)	337 100.0)		284 (100.0)	287 (100.0)	
BB	377 (66.0)	230 (68.2)	0.491	192 (67.6)	198 (69.0)	0.722
ACEi	104 (18.2)	50 (14.8)	0.190	59 (20.8)	48 (16.7)	0.215
ARB	311 (54.5)	184 (54.6)	0.969	154 (54.2)	158 (55.1)	0.843
ARNI	57 (10.0)	57 (16.9)	0.002	34 (12.0)	45 (15.7)	0.200
Nitrate	41 ((7.2)	33 (9.8)	0.223	21 (7.4)	26 (9.1)	0.310
MRA	141 (24.7)	121 (35.9)	0.001	81 (28.5)	94 (32.8)	0.221
SGLT2i	225 (39.4)	186 (55.2)	<0.001	130 (45.8)	147 (51.2)	0.193

Values are *n* (%), mean ± SD, or median (Q1-Q3).

ACEi, angiotensin-converting enzyme inhibitor; ACS, acute coronary syndrome; ARB, angiotensin receptor blocker; ARNI, angiotensin receptor–neprilysin inhibitor; BB, beta-blocker; BMI, body mass index; CABG, coronary artery bypass grafting; CAC, coronary artery calcification; COPD, chronic obstructive pulmonary disease; MI, myocardial infarction; MRA, mineralocorticoid receptor antagonist; NSTEMI, non–ST-segment elevation myocardial infarction; PCI, percutaneous coronary intervention; PSM, propensity score matching; SGLT2i, sodium-glucose cotransporter 2 inhibitor; STEMI, ST-segment elevation myocardial infarction.

### 3.2 Coronary lesion characteristics

In the overall cohort, 1,041 target lesions were analyzed (676 in the no or mild CAC group and 365 in the moderate-to-severe CAC group). After propensity score matching, 670 lesions remained (359 and 311, respectively). Patients with moderate-to-severe CAC exhibited significantly more complex coronary anatomy compared with those with no or mild CAC. They had higher SYNTAX scores (median 18.0 vs. 11.0; *p* < 0.001) and a greater prevalence of three-vessel disease (68.7% vs. 38.2%; *p* < 0.001). Other high-risk angiographic features, such as chronic total occlusions, bifurcations, tortuous segments, and ostial involvement, were also more frequently observed. These differences remained statistically significant after PSM.

On IVUS analysis, the main plaque composition in the moderate-to-severe CAC group was predominantly calcified (38.9%) or mixed (49.6%), whereas soft (46.0%) and fibrous (27.5%) plaques were more common in the no/mild CAC group. Additionally, the moderate-to-severe CAC group had greater maximum calcium arc and length, and more calcium nodules (37.5% vs. 4.9%). Detailed lesion characteristics and IVUS findings are summarized in Table 2.

**Table 2:** Coronary lesion characteristics on angiography and IVUS.

Variables	Before PSM			After PSM		
	No/mild calcification	Moderate/severe calcification	*P* value	No/mild calcification	Moderate/severe calcification	*P* value
Coronary artery characteristics (*n* = 914)						
Number of diseased coronary arteries						
1-vessel	172 (25.6)	30 (8.1)	<0.001	66 (22.9)	25 (8.7)	<0.001
2-vessel	184 (31.9)	79 (23.4)	0.006	94 (32.6)	75 (26.0)	0.082
3-vessel	220 (38.2)	229 (68.7)	<0.001	128 (44.4)	188 (65.3)	<0.001
Multi-vessel	404 (70.1)	308 (91.1)	<0.001	222 (77.1)	263 (91.3)	<0.001
LM disease	46 (8.0)	107 (31.7)	<0.001	23 (8.0)	86 (29.9)	<0.001
SYNTAX score	11.0 (7.0–16.0)	18.0 (12.0–25.5)	<0.001	12.0 (8.0–17.0)	17.8 (12.0–25.0)	<0.001
Angiographic lesion characteristics (*n* = 1,041)						
Target lesion site on coronary angiography						
LMCA	18 (2.7)	55 (15.1)	<0.001	9 (2.5)	42 (13.5)	<0.001
LAD	349 (51.6)	194 (53.2)	0.639	178 (49.6)	170 (54.7)	0.189
Ramus	1 (0.1)	1 (0.3)	1.000	1 (0.3)	1 (0.3)	1.000
LCx	101 (14.9)	33 (9.0)	0.007	54 (15.0)	28 (9.0)	0.017
RCA	207 (30.6)	82 (22.5)	0.006	117 (32.6)	70 (22.5)	0.004
Stenosis severity (%)	90.0 (80.0–95.0)	90.0 (80.0–90.0)	0.147	90.0 (80.0–95.0)	90.0 (80.0–90.0)	0.087
Lesion Type						
Type A	74 (10.9)	8 (2.2)	<0.001	36 (10.0)	7 (2.2)	<0.001
Type B1	212 (31.4)	41 (11.2)	<0.001	113 (31.5)	38 (12.2)	<0.001
Type B2	298 (44.1)	119 (32.6)	<0.001	152 (42.3)	105 (33.8)	0.023
Type C	92 (13.6)	197 (54.0)	<0.001	58 (16.2)	161 (51.8)	<0.001
Chronic total occlusion	17 (2.5)	19 (5.2)	0.023	11 (3.1)	16 (5.1)	0.172
Bifurcation lesion	27 (4.0)	55 (15.1)	<0.001	18 (5.0)	45 (14.5)	<0.001
Tortuous lesion	39 (5.8)	42 (11.5)	0.001	25 (7.0)	36 (11.6)	0.039
Ostial lesion	54 (8.0)	91 (24.9)	<0.001	28 (7.8)	76 (24.4)	<0.001
Presence of thrombus	226 (33.4)	55 (15.1)	<0.001	109 (30.4)	42 (13.5)	<0.001
Dissection	154 (22.8)	37 (10.1)	<0.001	66 (18.4)	31 (10.0)	0.002
TIMI Flow Grade						
TIMI 0	118 (17.5)	52 (14.2)	0.181	60 (16.7)	46 (14.8)	0.497
TIMI 1	65 (9.6)	23 (6.3)	0.067	38 (10.6)	18 (5.8)	0.025
TIMI 2	88 (13.0)	39 (10.7)	0.302	40 (11.1)	29 (9.3)	0.440
TIMI 3	405 (59.9)	251 (68.8)	0.005	221 (61.6)	218 (70.1)	0.020
Ivus-derived lesion characteristics						
Plaque characteristics						
Soft plaque	311 (46.0)	6 (1.6)	<0.001	150 (41.8)	5 (1.6)	<0.001
Fibrous plaque	186 (27.5)	36 (9.9)	<0.001	99 (27.6)	33 (10.6)	<0.001
Calcified plaque	0 (0.0)	142 (38.9)	<0.001	0 (0.0)	120 (38.6)	<0.001
Mixed plaque	179 (26.5)	181 (49.6)	<0.001	110 (30.6)	153 (49.2)	<0.001
Calcification severity						
None	435 (64.3)	0 (0.0)		217 (60.4)	0 (0.0)	
Mild	241 (35.7)	0 (0.0)		142 (39.6)	0 (0.0)	
Moderate	0 (0.0)	164 (44.9)		0 (0.0)	145 (46.6)	
Severe	0 (0.0)	201 (55.1)		0 (0.0)	166 (53.4)	
Maximum calcium arc (degrees)	95.0 (67.0–117)	288.5 (233.0–360.0)	<0.001	94.0 (67.0–120.0)	283.0 (231.0–360.0)	<0.001
Calcium length (mm)	5.3 (3.0–10.0)	19.0 (10.0–30.0)	<0.001	5.5 (3.0–10.7)	19.7 (10.2–30.6)	<0.001
Calcium distribution						
Superficial	212 (31.4)	280 (76.6)	<0.001	129 (35.9)	235 (75.6)	<0.001
Deep	13 (1.9)	0 (0.0)		7 (1.9)	0 (0.0)	
Both	22 (3.3)	83 (22.7)		10 (2.8)	74 (23.8)	
Calcium Nodule	33 (4.9)	137 (37.5)	<0.001	22 (6.1)	119 (39.3)	<0.001
Minimal lumen diameter (mm)	1.8 ± 0.3	1.8 ± 0.4	0.970	1.7 (1.5–2.0)	1.8 (1.5–1.9)	0.800
Minimal lumen area (mm^2^)	2.4 (1.9–3.0)	2.4 (2.0–2.9)	0.664	2.5 (1.9–3.0)	2.4 (2.0–2.9)	0.998
Plaque burden (%)	80.0 (75.0–84.0)	80.0 (76.0–83.0)	0.152	80.0 (75.0–84.0)	80.0 (76.0–83.0)	0.136
Distal reference EEM diameter (mm)	3.5 ± 0.7	3.4 ± 0.6	0.002	3.4 ± 0.6	3.4 ± 0.6	0.140
Distal reference lumen diameter (mm)	3.0 ± 0.5	2.9 ± 0.5	<0.001	3.0 ± 0.5	2.9 ± 0.5	0.001
Distal reference lumen area (mm^2^)	6.7 (5.3–8.8)	5.9 (4.8–7.6)	<0.001	6.5 (5.3–8.5)	6.0 (4.8–7.6)	0.001
Proximal reference EEM diameter (mm)	4.2 ± 0.6	4.3 ± 0.5	0.817	4.2 ± 0.5	4.3 ± 0.5	0.196
Proximal reference lumen diameter (mm)	3.8 ± 0.6	3.9 ± 0.7	0.003	3.8 ± 0.6	4.0 ± 0.7	0.001
Proximal reference lumen area (mm^2^)	10.5 (8.4–13.4)	11.4 (8.8–14.8)	0.005	10.4 (8.4–12.3)	11.6 (8.8–14.8)	0.002
Lesion length (mm)	33.0 (24.0–45.0)	45.0 (32.0–62.0)	<0.001	35.0 (26.0–45.0)	45.0 (32.0–60.6)	<0.001

Values are *n* (%), mean ± SD, or median (Q1–Q3).

IVUS, intravascular ultrasound; LAD, left anterior descending artery; LCx, left circumflex artery; LMCA, left main coronary artery; PSM, propensity score matching; RCA, right coronary artery.

### 3.3 Procedural characteristics

Patients with moderate-to-severe CAC underwent more intensive lesion preparation and device utilization compared with those in the no or mild CAC group. Predilation was performed more frequently (94.8% vs. 76.6%; *p* < 0.001), and the number of balloons used per lesion was higher (median 2.0 vs. 1.0; *p* < 0.001). Advanced lesion preparation techniques were employed significantly more often, including non-compliant balloons (55.9% vs. 15.8%), scoring balloons (65.2% vs. 11.4%), and atherectomy (7.7% vs. 0.1%) (all *p* < 0.001).

These patients also received longer and larger stents. Postdilation rates were similar (95.6%), but the moderate-to-severe CAC group required more balloons and tended to use larger sizes. All differences in procedural strategy and device use remained statistically significant after PSM. Detailed procedural characteristics are in Table 3.

**Table 3:** Procedural characteristics of percutaneous coronary intervention.

Variables	Before PSM			After PSM		
	No/mild calcification (*n* = 676)	Moderate/severe calcification (*n* = 365)	*P* value	No/mild calcification (*n* = 359)	Moderate/severe calcification (*n* = 311)	*P* value
Vascular access						
Radial	628 (92.9)	289 (79.2)	<0.001	327 (91.1)	250 (80.4)	<0.001
Femoral	41 (6.1)	69 (18.9)	<0.001	28 (7.8)	54 (17.4)	<0.001
Dual access	7 (1.0)	7 (1.9)	0.238	4 (1.1)	7 (2.3)	0.362
Guide catheter size						
6 Fr	575 (85.1)	231 (63.3)	<0.001	301 (83.8)	198 (63.7)	<0.001
7 Fr	101 (14.9)	134 (36.7)		58 (16.2)	113 (36.3)	
IABP	1 (0.1)	1 (0.3)	1.000	1 (0.3)	1 (0.3)	1.000
ECMO	0 (0.0)	0 (0.0)		0 (0.0)	0 (0.0)	1.000
Mechanical ventilation	10 (1.5)	7 (1.9)	0.594	8 (2.2)	6 (1.9)	0.787
Lesion preparation before stenting						
Pre-dilatation	581 (85.9)	346 (94.8)	<0.001	281 (78.3)	294 (94.5)	<0.001
Non-compliant balloon	107 (15.8)	204 (55.9)	<0.001	59 (16.4)	168 (54.0)	<0.001
Cutting balloon	0 (0.0)	4 (1.1)	0.015	0 (0.0)	4 (1.3)	0.046
Scoring balloon	77 (11.4)	144 (65.2)	<0.001	47 (13.1)	125 (40.2)	<0.001
Rotational atherectomy	1 (0.1)	28 (7.7)	<0.001	0 (0.0)	26 (8.4)	<0.001
Number of pre-dilatation balloons	1.0 (1.0–2.0)	2.0 (1.0–2.0)	<0.001	1.0 (1.0–1.0)	2.0 (1.0–2.0)	<0.001
Max diameter pre-dilatation balloon (mm)	2.5 (2.0–2.75)	2.75 (2.5–3.0)	<0.001	2.5 (2.0–2.8)	2.8 (2.5–3.0)	<0.001
Thrombus aspiration	22 (3.3)	3 (0.8)	0.011	10 (2.8)	2 (0.6)	0.041
Stent deployment						
Stent implantation	675 (99.9)	362 (99.2)	0.094	359 (100.0)	309 (99.4)	0.128
Drug-eluting stents	662 (97.9)	361 (98.9)	0.022	335 (98.9)	308 (99.0)	0.156
Bioresorbable stent	13 (1.9)	1 (0.3)		4 (1.1)	1 (0.3)	
Number of stents	1.0 (1.0–1.0)	1.0 (1.0–2.0)	<0.001	1.0 (1.0–1.0)	1.0 (1.0–2.0)	<0.001
Max stent diameter (mm)	3.0 (3.0–3.5)	3.5 (3.0–3.5)	0.232	3.0 (3.0–3.5)	3.5 (3.0–3.5)	0.033
Total stent length (mm)	36.0 (28.0–48.0)	48.0 (38.0–67.0)	<0.001	38.0 (28.0–48.0)	48.0 (38.0–66.0)	<0.001
Post-dilatation						
Post-dilatation	646 (95.6)	349 (95.6)	0.968	346 (96.4)	299 (96.1)	0.872
Number of post-dilatation balloons	1.0 (1.0–2.0)	2.0 (1.0–3.0)	<0.001	1.0 (1.0–2.0)	2.0 (1.0–2.0)	<0.001
Max diameter post-dilatation balloon (mm)	3.5 (3.25–4.0)	3.75 (3.5–4.5)	0.009	3.5 (3.3–4.0)	3.8 (3.5–4.5)	0.005

Values are *n* (%), mean ± SD, or median (Q1–Q3).

ECMO, extracorporeal membrane oxygenation; IABP, intra-aortic balloon pump; PCI, percutaneous coronary intervention; PSM, propensity score matching.

### 3.4 Procedural outcomes

Procedural success was lower in the moderate-to-severe CAC group than in the no or mild CAC group, both before (79.2% vs. 94.8%) and after PSM (81.0% vs. 93.9%; *p* < 0.001 for both). IVUS assessment showed lower optimal stent deployment in the moderate-to-severe group (64.4% vs. 78.8% before matching; 65.9% vs. 77.7% after matching; *p* < 0.001 and *p* = 0.001).

Fewer patients in the moderate-to-severe CAC group achieved the recommended minimum stent area (MSA). Specifically, the proportion achieving MSA ≥5.0 mm^2^ was significantly lower (72.6% vs. 80.4%; *p* = 0.016), as was the proportion achieving MSA ≥5.5 mm^2^ (60.0% vs. 69.8%; *p* = 0.008). In contrast, major edge dissection, significant malapposition, and edge plaque burden >50% were low and similar between groups. Procedural complications and in-hospital adverse events were uncommon and comparable. Details are in Table 4.

**Table 4:** Procedural and 1-year clinical outcomes.

Variables	Before PSM			After PSM		
	No/mild calcification (*n* = 676)	Moderate/severe calcification (*n* = 365)	*P* value	No/mild calcification (*n* = 359)	Moderate/severe calcification (*n* = 311)	*P* value
Angiographic success	641 (94.8)	289 (79.2)	<0.001	337 (93.9)	252 (81.0)	<0.001
IVUS findings						
Major stent malapposition	9 (1.3)	7 (1.9)	0.185	5 (1.4)	4 (1.3)	0.312
Stent edge dissection	11 (1.6)	6 (1.6)	0.245	7 (1.9)	4 (1.3)	0.252
Plaque burden at stent edges >50%	48 (7.1)	30 (8.3)	0.494	25 (7.0)	28 (9.1)	0.317
Tissue protrusion	56 (8.3)	19 (5.2)	0.048	27 (7.5)	16 (5.1)	0.147
Met minimum stent area criteria	572 (84.7)	254 (70.2)	<0.001	301 (83.8)	222 (71.8)	<0.001
Minimum stent area ≥ 5.0 mm^2^	556 (82.5)	260 (71.8)	<0.001	288 (80.4)	225 (72.6)	0.016
Minimum stent area ≥ 5.5 mm^2^	498 (73.9)	214 (59.1)	<0.001	250 (69.8)	186 (60.0)	0.008
Minimum stent area (mm^2^)	6.8 (5.4–8.8)	5.8 (4.9–7.5)	<0.001	6.6 (5.3–8.4)	5.8 (4.9–7.5)	<0.001
Relative stent expansion (%)	100.0 (95.0–10.7.0)	100.0 (92.0–105.0)	0.004	100.0 (94.0–107.0)	100.0 (92.0–105.0)	0.278
Relative expansion >90%	595 (88.3)	289 (79.8)	<0.001	313 (87.4)	255 (82.3)	0.062
Optimal stent implantation	533 (78.8)	235 (64.4)	<0.001	279 (77.7)	205 (65.9)	0.001
Procedure-related complications						
Coronary perforation	3 (0.4)	5 (1.5)	0.154	3 (0.4)	4 (1.1)	0.248
Cardiac tamponade	2 (0.3)	3 (0.8)	0.241	2 (0.3)	3 (0.8)	0.351
Coronary dissection	12 (1.8)	8 (2.2)	0.640	12 (1.8)	8 (2.2)	0.640
Side branch loss	15 (2.2)	14 (3.8)	0.130	15 (2.2)	14 (3.8)	0.130
In-hospital outcomes						
MACE	11 (1.9)	7 (2.1)	0.865	9 (3.1)	6 (2.1)	0.433
All-cause death	5 (0.9)	1 (0.3)	0.301	4 (1.4)	1 (0.3)	0.373
MI	4 (0.7)	6 (1.8)	0.129	3 (1.0)	5 (1.7)	0.725
Stent thrombosis	1 (0.2)	0 (0.0)	1.000	1 (0.3)	0 (0.0)	1.000
TLR	1 (0.2)	0 (0.0)	1.000	1 (0.3)	0 (0.0)	1.000
Stroke	3 (0.5)	0 (0.0)	0.300	3 (1.0)	0 (0.0)	0.249
1-year outcomes						
MACE	24 (4.2)	32 (9.5)	0.001	18 (6.3)	27 (9.4)	0.162
All-cause death	18 (3.1)	22 (6.5)	0.016	14 (4.9)	19 (6.6)	0.370
Cardiac death	14 (2.4)	19 (5.7)	0.012	12 (4.2)	16 (5.6)	0.427
MI	5 (0.9)	12 (3.6)	0.004	3 (1.0)	10 (3.5)	0.089
Stent thrombosis	3 (0.5)	2 (0.6)	1.000	2 (0.7)	2 (0.7)	1.000
TLR	4 (0.7)	5 (1.5)	0.246	3 (1.0)	5 (1.7)	0.725
Stroke	6 (1.0)	1 (0.3)	0.270	5 (1.7)	0 (0.0)	0.061

Values are *n* (%), mean ± SD, or median (Q1–Q3).

IVUS, intravascular ultrasound; MACE, major adverse cardiac events; MI, myocardial infarction; PCI, percutaneous coronary intervention; PSM, propensity score matching; TLR, target lesion revascularization.

### 3.5 1-Year outcomes

Before matching, 1-year MACE was higher in moderate-to-severe CAC than in no or mild CAC (9.5% vs. 4.3%; *p* = 0.001). After PSM, this difference persisted numerically (9.4% vs. 6.3%) but was no longer statistically significant (*p* = 0.162) (Table 4). A similar pattern was observed for individual MACE components: differences in all-cause death, cardiac death, and myocardial infarction were significant before matching but attenuated post-matching. Rates of target lesion revascularization, stent thrombosis, and stroke remained low and comparable between groups (Table 4).

**Figure 3:**
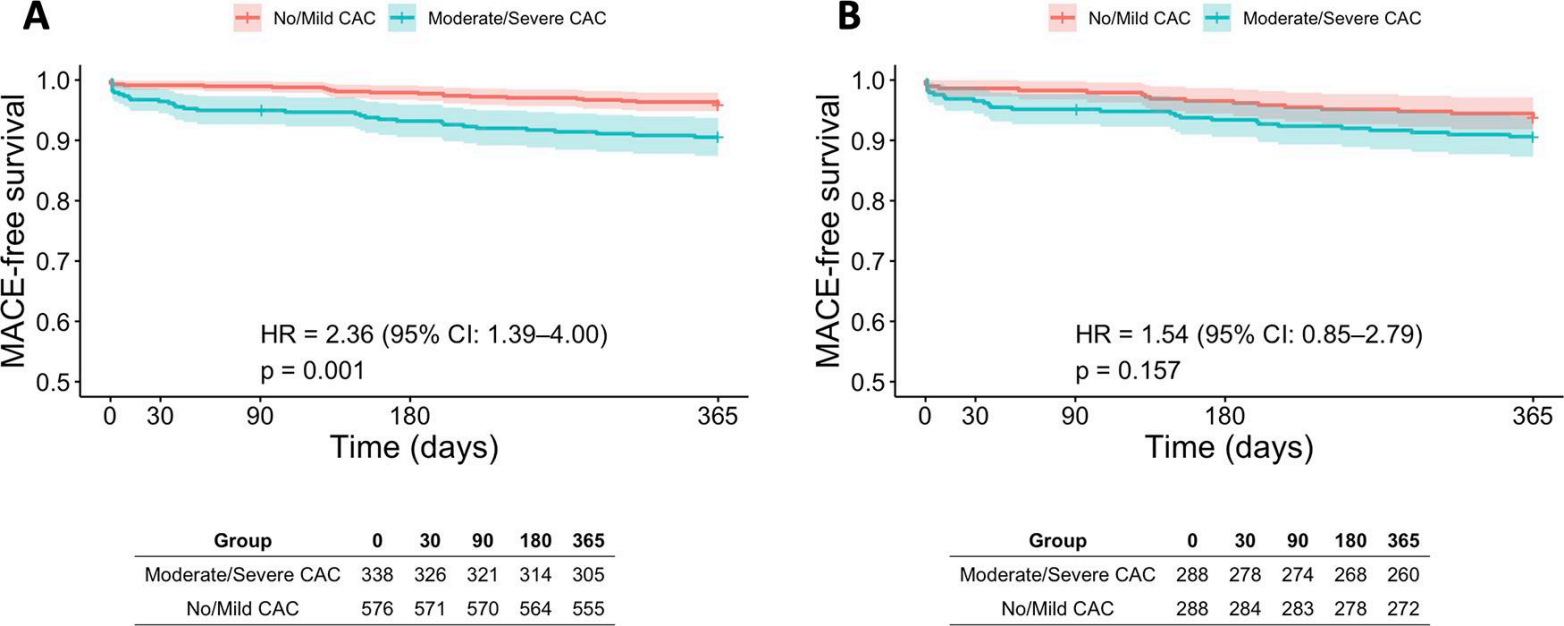
Kaplan–Meier curves for 1-year MACE before **(A)** and after **(B)** propensity score matching. **(A)** Before PSM, patients with moderate-to-severe CAC had a significantly higher incidence of MACE compared with those with none/mild CAC (9.5% vs. 4.2%; HR: 2.36, 95% CI: 1.39–4.00; *p* = 0.001). **(B)** After PSM, the difference was attenuated and no longer statistically significant (9.4% vs. 6.3%; HR: 1.54, 95% CI: 0.85–2.79; *p* = 0.157). CAC, coronary artery calcification; CI, confidence interval; HR, hazard ratio; IVUS, intravascular ultrasound; MACE, major adverse cardiac events; PCI, percutaneous coronary intervention; PSM, propensity score matching.

In the matched cohort, univariable Cox regression confirmed no significant association between moderate-to-severe CAC and 1-year MACE (HR 1.538; 95% CI 0.847–2.792; *p* = 0.157) (Table 5, Figure 3). In contrast, adverse outcomes were associated with older age, chronic kidney disease, acute heart failure, reduced left ventricular ejection fraction, and suboptimal IVUS-defined stent implantation (Supplementary Table 5). Results from exploratory multivariable analyses in the unmatched cohort are provided in Supplementary Result 3, Supplementary Table 6. Exploratory stratified analyses suggested that the clinical impact of IVUS-defined stent optimization varied according to CAC severity, with adverse outcomes largely confined to patients with moderate-to-severe calcification. Full details of these analyses are provided in the Supplementary Material (Supplementary Result 4, Supplementary Table 7, Supplementary Figure 1).

**Table 5:** Univariate analysis of the association between moderate-to-severe coronary calcification and adverse cardiac outcomes.

Variables	Before PSM			After PSM		
	HR	95% CI	*P* value	HR	95% CI	*P* value
MACE	2.357	1.389–4.002	0.001	1.538	0.847–2.792	0.157
All-cause death	2.166	1.162–4.038	0.015	1.394	0.699–2.780	0.346
Cardiac death	2.402	1.205–4.791	0.013	1.370	0.648–2.897	0.409
MI	4.200	1.480–11.922	0.007	3.386	0.932–12.303	0.064
TLR	2.235	0.596–8.261	0.235	1.711	0.409–7.162	0.462

CI, confidence interval; HR, hazard ratio; MACE, major adverse cardiac events; MI, myocardial infarction; PSM, propensity score matching; TLR, target lesion revascularization.

## 4 Discussion

This study evaluated the prognostic relevance of moderate-to-severe CAC in patients undergoing IVUS-guided PCI. Moderate-to-severe CAC was observed in 37.0% of patients. Before PSM, these patients exhibited significantly higher 1-year rates of MACE. However, after matching, the difference was no longer statistically significant, and univariable Cox analysis confirmed the absence of an independent association between moderate-to-severe CAC and 1-year MACE. These findings suggest that, in the era of intravascular imaging and systematic lesion preparation, the adverse prognostic impact historically attributed to CAC may be substantially attenuated.

The prevalence of moderate-to-severe CAC identified by IVUS (37.0%) exceeded angiography-based estimates (18%–30%) (1–3). This likely reflects the superior sensitivity of intravascular imaging in detecting and characterizing calcium, as well as the inclusion of a high-risk PCI population. Indeed, fluoroscopic angiography identified calcification in only 23.0% of lesions, underscoring its limited diagnostic yield. Collectively, these observations emphasize the essential role of intravascular imaging in accurate calcium detection, risk stratification, and procedural planning.

A key challenge in PCI for CAC is the need for meticulous lesion preparation, especially when the calcium arc exceeds 180° or extends over ≥5 mm (18). In our study, preparation was intensive, protocol-driven, and guided by iterative IVUS assessment. Predilation was performed in 94.8% of patients, with frequent use of non-compliant balloons (55.9%), scoring balloons (65.2%), and rotational atherectomy (7.7%). Lesion preparation intensity appeared to be primarily driven by IVUS-defined calcium burden rather than luminal narrowing or thrombus. A recent analysis at our center showed that among those undergoing atherectomy, 67.7% received it as a primary rather than bailout strategy. Compared with prior reports, our rates of lesion modification were substantially higher, reflecting a center-specific protocol that allowed real-time evaluation and therapeutic escalation (5, 19, 20). This approach likely contributed to improved device deliverability and stent expansion.

New-generation DES were used in 99.0% of cases. Patients with moderate-to-severe CAC underwent more extensive stenting, as evidenced by a higher stent count (median 2.0 vs. 1.0), longer total stent length (48.0 mm vs. 38.0 mm), and larger maximal stent diameter (3.5 mm vs. 3.0 mm). These differences likely stem from both the greater anatomical complexity of calcified disease and the use of IVUS to optimize stent sizing and positioning. Consistent with prior studies, contemporary data support the clinical benefit of newer-generation DES in patients with calcified and complex coronary lesions (19–22).

In line with this intensive strategy, postdilation was performed in 96.6% of cases, a rate substantially higher than previously reported by Jia et al. (70.3%), Hemetsberger et al. (59.1%), and Doan et al. in the CAPIRO study (77.7%) (5, 19, 20). In calcified lesions, larger balloons (median 3.75 mm) were selected to approximate proximal reference vessel dimensions derived from IVUS. These findings underscore a paradigm shift in PCI, from merely delivering a stent to systematically achieving optimal stent expansion and apposition (23, 24).

Intravascular imaging–guided optimization has been shown to reduce the risk of stent thrombosis and restenosis (13, 25–27). In our study, optimal stent implantation by IVUS criteria was achieved in 64.4% of patients. The main cause of suboptimal outcomes was inadequate expansion in heavily calcified segments, reflected by lower MSA attainment in the moderate-to-severe CAC group (70.2% vs. 84.7%). Other optimization metrics, apposition (98.1%), absence of edge dissection (98.0%), and low plaque burden at stent margins (92.5%), remained high and comparable between groups. These findings confirm calcium as the main barrier to full stent expansion despite aggressive preparation. Our optimization rates compare favorably with prior trials: 53% in ULTIMATE, 54% in IVUS-XPL, 56.8% for CAC in OPTIVUS, and 55.5% in RENOVATION (13, 25, 26, 28). In the CAPIRO registry, only 41.5% of calcified lesions reached the IVUS MSA target ≥5.5 mm^2^ (19). Collectively, these results underscore the technical challenges of CAC treatment and the value of a structured, imaging-guided PCI strategy.

Historically, moderate-to-severe CAC was regarded as an independent predictor of adverse outcomes after PCI (1, 4–6). This paradigm, however, is increasingly challenged by contemporary data. Recent studies, including CAPIRO (1.09%), Wongpraparut et al. (4.3%), and Zhang et al. (7.5%), have reported substantially lower MACE rates in patients with calcified lesions compared with earlier cohorts (19, 29, 30). Moreover, several analyses have demonstrated that CAC is no longer an independent predictor of adverse outcomes after multivariable adjustment, suggesting that procedural technique and patient comorbidities may exert greater prognostic influence than calcification alone (1, 5, 20).

Our findings are consistent with this evolving perspective. After propensity score adjustment, moderate-to-severe CAC was not independently associated with 1-year MACE. Instead, adverse outcomes were more closely linked to clinical comorbidities and suboptimal stent implantation as assessed by IVUS. Notably, the composition of adverse events appears to have shifted. Previously dominant complications, including target lesion revascularization and stent thrombosis, have substantially declined, thereby narrowing the outcome gap between calcified and non-calcified lesions. Thus, with systematic lesion preparation, intravascular imaging, and contemporary medical therapy, the adverse prognostic impact of CAC appears attenuated. Although moderate-to-severe CAC correlated with more complex anatomy and lower rates of optimal stent expansion, its lack of independent association with MACE indicates that prognosis is now determined primarily by procedural success, with CAC functioning chiefly as a marker of technical complexity.

This study has several strengths. It reflects real-world practice at a high-volume PCI center, with all procedures performed under IVUS guidance using a standardized stepwise protocol. PSM was applied to minimize baseline confounding, thereby enhancing internal validity. Nonetheless, several limitations merit consideration. First, this was a single-center study without a comparator group lacking IVUS guidance, which limits the ability to assess the incremental benefit of imaging. Second, calcium severity was determined visually by operators without core laboratory adjudication, potentially introducing bias. Third, advanced calcium modification devices, such as intravascular lithotripsy or excimer laser, were unavailable during the study period, which may have constrained procedural options. In patients with acute coronary syndrome, intracoronary thrombus may have influenced soft plaque evaluation and procedural strategy, with limited impact on IVUS-derived calcification parameters. In addition, the 1-year follow-up provides only intermediate-term outcomes and does not capture quality-of-life measures. Finally, the relatively low event rate may have reduced statistical power to detect modest associations.

## 5 Conclusions

In this IVUS-guided PCI cohort, moderate-to-severe coronary calcification was not significantly associated with 1-year MACE after propensity score matching. These findings indicate that, in contemporary practice where IVUS-guided PCI with systematic lesion preparation and stent optimization is routinely applied, the adverse prognostic impact historically attributed to coronary calcification appears attenuated. Larger, multicenter studies are warranted to validate these observations and to further clarify the role of intravascular imaging in the management of calcified coronary lesions.

## Data Availability

The raw data supporting the conclusions of this article will be made available by the authors, without undue reservation.
