# Supplementary material for: Impact of moderate-to-severe coronary calcification on 1-year clinical outcomes after IVUS-guided PCI
Source: Front Cardiovasc Med. 2026 Feb 6;13:1720958. doi: 10.3389/fcvm.2026.1720958 (PMC12920530; doi:10.3389/fcvm.2026.1720958)
Supplement: Supplementary file 1 [file Datasheet1.docx]

Supplementary Material

Impact of moderate-to-severe coronary calcification on 1-year clinical outcomes after IVUS-guided PCI

Thanh Cong Nguyen^1^, Vu Hoang Vu^1,2*^, Bao Thien Duong^1^, Hoa Tran^1,2^, Khoi Minh Le^2,3^, Hung Minh Ngo^4^, Van Hoang^5^, Thai Quoc Nguyen^6^, Binh Quang Truong^1,2^

^1^Interventional Cardiology Department, University Medical Center Ho Chi Minh City, Ho Chi Minh City, Vietnam

^2^School of Medicine, University of Medicine and Pharmacy at Ho Chi Minh City, Ho Chi Minh City, Vietnam

^3^Cardiac Imaging Unit, University Medical Center Ho Chi Minh City, Ho Chi Minh City, Vietnam

^4^Interventional Cardiology Department, Cho Ray Hospital, Ho Chi Minh City, Vietnam

^5^Hanoi Heart Hospital, Hanoi City, Vietnam

^6^Vietnam National Heart Institute, Bach Mai Hospital, Hanoi City, Vietnam

* Correspondence:
Vu Hoang Vu
vu.vh@umc.edu.vn

**Table of contents**

[Supplementary appendix 1. Methods 4](#_Toc217041105)

[Supplementary method 1. Post-PCI patient management program 4](#_Toc217041106)

[Supplementary method 2. Eligibility criteria 5](#_Toc217041107)

[Supplementary method 3. Calcification assessment 6](#_Toc217041108)

[Supplementary method 4. IVUS-guided PCI procedure 7](#_Toc217041109)

[Supplementary method 5. IVUS criteria for stent optimization 9](#_Toc217041110)

[Supplementary method 6. Definition of variables 10](#_Toc217041111)

[Supplementary method 7. Sample size estimation 13](#_Toc217041112)

[Supplementary method 8. STROBE checklist for observational cohort studies 14](#_Toc217041113)

[Supplementary appendix 2. Additional results 17](#_Toc217041114)

[Supplementary result 1. Intra- and Inter-Observer Reproducibility of IVUS-Based Calcification Assessment 17](#_Toc217041115)

[Supplementary result 2. Sensitivity and interaction analyses addressing residual confounding by peripheral artery disease and renal replacement therapy 18](#_Toc217041116)

[Supplementary result 3. Multivariable Cox regression analysis of factors associated with 1-year MACE 19](#_Toc217041117)

[Supplementary result 4. Impact of IVUS-defined stent optimization stratified by CAC severity 20](#_Toc217041118)

[Supplementary result 5. Impact of ACS Status on the Association Between CAC Severity and 1-Year MACE 21](#_Toc217041119)

[Supplementary tables 22](#_Toc217041120)

[Supplementary table 1. Intra- and inter-observer reproducibility of categorical IVUS-based coronary calcification grading (n = 60) 22](#_Toc217041121)

[Supplementary table 2. Intra- and inter-observer reproducibility of quantitative IVUS-based coronary calcification measurements (n = 60) 23](#_Toc217041122)

[Supplementary table 3. Sensitivity and interaction analyses evaluating residual confounding and effect modification by peripheral artery disease and renal replacement therapy 24](#_Toc217041124)

[Supplementary table 4. Laboratory characteristics 26](#_Toc217041125)

[Supplementary table 5. Univariable Cox regression for 1-year MACE 28](#_Toc217041127)

[Supplementary table 6. Multivariable Cox regression analysis of factors associated with 1-year MACE 30](#_Toc217041128)

[Supplementary table 7. Association between IVUS-defined stent optimization and 1-year MACE stratified by coronary artery calcification severity 32](#_Toc217041129)

[Supplementary table 8. Impact of ACS status on the association between moderate-to-severe CAC and 1-Year MACE 33](#_Toc217041130)

[Supplementary Figures 35](#_Toc217041131)

**Abbreviations:**

CAC: coronary artery calcification

CI: confidence interval

HR: hazard ratio

IVUS: intravascular ultrasound

MACE: major adverse cardiac events

MI: myocardial infarction

MSA: minimum stent area

PCI: percutaneous coronary intervention

# Supplementary appendix 1. Methods

# Supplementary method 1. Post-PCI patient management program

At University Medical Center Ho Chi Minh City, a structured, multidisciplinary post–percutaneous coronary intervention (PCI) management program has been implemented to enhance secondary prevention and long-term outcomes. The program integrates standardized follow-up schedules, nurse-led education, digital health tools, and real-time data management.

***Multidisciplinary team and nurse roles***

The program is delivered by a multidisciplinary team of interventional cardiologists, nurses, pharmacists, dietitians, and general practitioners. Nurses play a pivotal role in in-hospital education, discharge planning, lifestyle counseling, and structured follow-up. All participating nurses receive structured training in cardiovascular pharmacology, secondary prevention, patient engagement, and telehealth communication.

***Patient education and self-monitoring***

Prior to discharge, patients attend standardized education sessions emphasizing cardiovascular risk control, physical activity, medication adherence, and symptom recognition. A patient handbook is provided to facilitate monitoring of vital signs, anginal symptoms, and daily health concerns, ensuring continuity between inpatient care and outpatient follow-up.

***Outpatient follow-up and digital infrastructure***

Patients are scheduled for follow-up at 1, 3, 6, and 12 months. Appointment reminders are sent via phone calls or mobile messages. At each contact, nurses assess symptoms, adherence, and adverse effects, and record findings in the REDCap platform, a secure, web-based research database. During clinic visits, cardiologists evaluate patient status and optimize guideline-directed medical therapy. Medication reconciliation, laboratory testing, and risk reassessment are routinely performed.

***Data standardization and registry integration***

All patients are enrolled in a PCI registry (ClinicalTrials.gov Identifier: NCT06071741), and data collection adheres to EuroHeart PCI data standards. Variables include demographics, procedural details, discharge therapy, and clinical outcomes, enabling real-time monitoring, inter-center benchmarking, and quality improvement initiatives.

# Supplementary method 2. Eligibility criteria

***Inclusion criteria***

Eligible participants were adults (≥18 years) diagnosed with coronary artery disease, including chronic coronary syndrome and acute coronary syndromes, such as ST-elevation myocardial infarction, non–ST-elevation myocardial infarction, or unstable angina. All patients underwent PCI according to current guideline-directed indications.

Patients with complex coronary anatomy (e.g., left main disease or multivessel coronary artery disease) underwent multidisciplinary counseling regarding revascularization options, and PCI was performed after multidisciplinary decision-making, informed consent, and confirmation of lesion suitability.

Intravascular ultrasound (IVUS) imaging was required for all enrolled patients. Based on IVUS findings, patients were stratified into two groups:

- No or mild calcification.
- Moderate-to-severe calcification.

In cases involving multiple target lesions or multivessel PCI during a single hospitalization, group assignment was determined by the lesion with the highest degree of calcification.

Calcification severity was defined exclusively by IVUS criteria:

- Severe calcification: calcium arc >270° extending ≥5 mm in length, or concentric (360°) calcification.
- Moderate calcification: calcium arc >180° on at least one frame without meeting severe criteria.

***Exclusion criteria***

Patients were excluded if they met any of the following:

- Absence of IVUS or suboptimal image quality precluding reliable lesion or post-stent assessment.
- Target lesion located in in-stent restenosis or a bypass graft.
- Hemodynamic instability or cardiogenic shock (SCAI stage C or higher) at the time of PCI.
- Contraindications or known allergy to antithrombotic therapy (e.g., heparin, aspirin, clopidogrel, ticagrelor, prasugrel).
- Estimated life expectancy <12 months.
- Pregnancy or breastfeeding.
- Refusal to participate or anticipated inability to complete 1-year follow-up.

# Supplementary method 3. Calcification assessment

Calcification assessment was performed according to a predefined and standardized institutional protocol, developed in accordance with contemporary international consensus documents on intracoronary imaging.^1,2^ Specifically, the assessment focused on calcium arc, length, depth, qualitative thickness markers, and the presence of calcified nodules.

***Calcium arc measurement***

The maximal calcium arc was measured using electronic angle tools centered at the geometric lumen axis (excluding the IVUS catheter artifact). Calcification severity was classified as follows:

- None/mild: calcium arc <180°.
- Moderate: arc ≥180° and <270°.
- Severe: arc ≥270° with longitudinal extension >5 mm, or 360° circumferential calcium.

Patients were grouped based on the most severely calcified lesion observed during the index procedure.

***Calcium length***

Calcium length was calculated from automated IVUS pullback by multiplying pullback speed by the duration of continuous calcium presence. Segments >5 mm were classified as extensive and considered for lesion preparation. Measurements were not performed during manual pullback but obtained from subsequent automated pullback runs.

***Calcium depth and qualitative thickness assessment***

Calcium depth was determined by the position of acoustic shadowing within the plaque.

- Superficial calcium: shadowing confined to the superficial 50% of the plaque plus media.
- Deep calcium: shadowing extending into the deeper 50% of the plaque and beyond the media.

Because IVUS cannot directly measure calcium thickness, surrogate markers were used. Reverberation artifacts (concentric echoes beyond the calcium interface) and a smooth calcium-lumen border were suggestive of thinner calcium (<0.5 mm), whereas irregular margins and absence of reverberation indicated thicker calcium (>0.5 mm).

***Calcified nodules***

Calcified nodules were identified by protrusion into the lumen with a convex leading edge and posterior acoustic shadowing. When present, they were recorded as high-risk features linked to stent deformation, underexpansion, and adverse long-term outcomes.

Calcified nodules were recorded as a separate morphological feature and were not used for CAC grading, which was determined by the highest degree of adjacent sheet-like calcification.

# Supplementary method 4. IVUS-guided PCI procedure

All PCI procedures were performed using standard techniques under IVUS guidance. Procedures were conducted by a trained team consisting of two interventional cardiologists, one dedicated nurse, and one certified cardiovascular technician.

***Equipment***

IVUS imaging was performed using either the POLARIS Multi-Modality Guidance System (Boston Scientific, USA) with OPTICROSS™ (40 MHz, 3.1 Fr) or OPTICROSS™ HD (60 MHz, 3.1 Fr) catheters, or the Philips Volcano platform (Philips, USA) with the Revolution® high-frequency rotational IVUS catheter (45 MHz, 3.2 Fr), depending on availability.

Despite differences in transducer frequency and catheter design, all IVUS systems used in this study provide a comparable effective penetration depth of approximately 5–6 mm, which is sufficient to visualize the full thickness of the coronary vessel wall, including both superficial and deep calcified plaques.

The pullback system, connection unit, and IVUS console were calibrated prior to each procedure.

**IVUS imaging protocol**

System preparation: The IVUS catheter was connected to the console, purged of air, and tested in a quality check mode to ensure a circular and uniform image.

Intracoronary vasodilation: Intracoronary nitroglycerin (100 – 200 µg) was administered to minimize vasospasm prior to catheter advancement.

Catheter advancement: The IVUS catheter was advanced at least 10 mm beyond the distal edge of the target lesion, using the guidewire track. In cases of tight stenosis, chronic total occlusion, or severe calcification, small-balloon predilatation was permitted to facilitate catheter crossing.

Pullback technique: Automated pullback (preferred) was performed at 0.5 mm/s. Manual pullback was reserved for tortuous or complex lesions. The entire lesion and reference segments were imaged.

***Pre-PCI IVUS assessment***

Pre-intervention imaging was used to:

- Evaluate plaque morphology (fibrotic, lipid-rich, calcified, mixed)
- Identify ulceration, dissection, thrombus, or calcium nodules
- Measure lesion length, minimum lumen area (MLA), plaque burden, and reference vessel diameters.
- Quantify calcium arc, length, and distribution for potential lesion preparation

***Lesion preparation***

In cases of moderate-to-severe calcification, lesion preparation was performed at the operator’s discretion using non-compliant balloons, scoring/cutting balloons, or rotational atherectomy. The latter was used either as a primary strategy or bailout when balloon or catheter passage was unsuccessful or calcium nodules were encountered.

***Stent selection under IVUS guidance***

Stent sizing followed a standardized IVUS-guided protocol. Stent diameter was primarily selected based on the distal reference vessel diameter. When lumen-based measurements were used, the stent diameter was rounded up to the nearest 0.25 mm; when EEM-based measurements were used, it was rounded down to the nearest 0.25 mm to avoid distal oversizing. Stent length was selected to ensure complete lesion coverage, including adjacent segments with significant plaque burden, while accounting for vessel tapering and lesion length.

***Post-stenting IVUS assessment***

After stent deployment, repeat IVUS was performed to assess:

- Minimum stent area (MSA) and relative expansion compared to distal reference.
- Stent apposition to the vessel wall.
- Presence of edge dissection, geographic miss, plaque burden at stent edges, and tissue prolapse.

# Supplementary method 5. IVUS criteria for stent optimization

Stent optimization was confirmed by IVUS when all of the following criteria were fulfilled, in accordance with established expert consensus documents and major IVUS-guided PCI studies:^1-3^

***Minimum stent area (MSA)***

The MSA was considered acceptable when meeting one of the following thresholds, according to reference vessel size:

- ≥5.0 mm² for vessels with reference diameter 3.0–3.5 mm.
- ≥5.5 mm² for vessels with reference diameter ≥3.5 mm.

Special anatomical sites:

- Left circumflex ostium: ≥6.0 mm².
- Left anterior descending ostium: ≥8.0 mm².
- Left main coronary artery: ≥10.0 mm².

***Relative expansion***

Relative expansion ≥90%, defined as: MSA / distal reference lumen area × 100.

***Adequate stent apposition***

Stent–vessel wall distance <0.4 mm, with any malapposed segment <1.0 mm in length.

***Edge plaque burden***

Plaque burden within 5 mm proximal and distal to the stent edge was <50%

***Edge dissection***

Dissections at the stent edge were acceptable only if limited to the intimal layer, with length <2.0 mm and circumferential extent <60°.

***Tissue protrusion***

Tissue protrusion was defined as tissue or plaque extrusion through the stent struts into the lumen on post-stent IVUS imaging and was recorded as an ancillary IVUS finding, not included as a criterion for optimal stent implantation.

# Supplementary method 6. Definition of variables

***Angiographic success***

Angiographic success was defined as achieving all of the following criteria at the target lesion site:

- Residual stenosis ≤30%, as visually estimated.
- Final TIMI flow grade 3.
- Preservation of all major side branches.
- Absence of major intraprocedural complications, including coronary perforation, acute vessel closure, or periprocedural death.

***All-cause mortality, cardiac death, and non-cardiac death***

- Cardiac death was defined as any death directly or indirectly attributable to cardiac causes, including myocardial infarction, heart failure, fatal arrhythmia, sudden cardiac death, unexplained death, or death related to cardiac procedures (e.g., PCI or cardiac surgery).
- Non-cardiac death was defined as any death clearly unrelated to cardiac causes, including malignancy, infection, trauma, suicide, pulmonary disease (e.g., pneumonia, pulmonary embolism), neurologic events (e.g., ischemic stroke, intracranial hemorrhage), or non-cardiac vascular conditions (e.g., ruptured aortic aneurysm).
- All-cause mortality encompassed all deaths, regardless of etiology

***Myocardial infarction***

Myocardial infarction (MI) was defined according to the Fourth Universal Definition of MI, requiring a rise and/or fall of cardiac troponin with at least one value above the 99th percentile upper reference limit, together with at least one of the following:

- Symptoms of myocardial ischemia
- New ischemic ECG changes (e.g., ST–T changes or new left bundle branch block)
- Development of pathological Q waves
- Imaging evidence of new loss of viable myocardium or new regional wall motion abnormality
- Identification of coronary thrombus by angiography or autopsy

Myocardial infarction was classified into the following subtypes:

- Type 1: Spontaneous MI related to atherosclerotic plaque rupture, ulceration, erosion, or dissection with resulting coronary thrombosis and ischemia.
- Type 2: MI secondary to an imbalance between myocardial oxygen supply and demand, unrelated to coronary thrombosis.
- Type 3: Cardiac death with suggestive symptoms or ECG changes before biomarker confirmation.
- Type 4a: MI related to PCI.
- Type 4b: MI due to stent thrombosis.
- Type 5: MI related to coronary artery bypass grafting.

Periprocedural MI (Type 4a) was defined as an elevation of cardiac troponin >5 times the 99th percentile URL in patients with normal baseline values, or a >20% increase in patients with elevated but stable or falling baseline values, along with at least one of the following:

- Symptoms of myocardial ischemia.
- New ischemic ECG changes.
- Imaging evidence of new loss of viable myocardium or regional wall motion abnormality.
- Angiographic evidence of procedural complication (e.g., dissection, occlusion, no-reflow).

***Stent thrombosis***

Stent thrombosis was defined and classified according to the Academic Research Consortium (ARC) criteria, based on both diagnostic certainty and timing relative to the index PCI procedure.

Definite stent thrombosis required angiographic or pathologic evidence of thrombus within the stent or within 5 mm of its edges, accompanied by at least one of the following clinical features occurring within 48 hours:

- Symptoms suggestive of myocardial ischemia.
- New ischemic ECG changes.
- Rise or fall in cardiac troponin levels.

Probable stent thrombosis was defined as:

- Unexplained sudden cardiac death within 30 days of the index procedure.
- Target-vessel myocardial infarction without a clear non-coronary cause.

Stent thrombosis was further categorized by timing as:

- Acute (0–24 hours post-PCI).
- Subacute (>24 hours to 30 days).
- Late (>30 days to 1 year).
- Very late (>1 year).

***Target lesion and target vessel revascularization***

Ischemia-driven revascularization was defined as any repeat PCI or coronary artery bypass grafting performed due to recurrent or persistent symptoms of myocardial ischemia.

- Target lesion revascularization (TLR): Repeat revascularization at the previously stented segment, including the stent and 5 mm proximal or distal to its edges.
- Target vessel revascularization (TVR): Any revascularization within the treated vessel, including the target lesion and other segments or side branches of the same vessel.
- Non-target vessel revascularization (non-TVR): Revascularization in a coronary artery other than the one treated during the index procedure.

***Stroke***

Stroke was defined as a clinical syndrome characterized by the sudden onset of a focal neurological deficit lasting ≥24 hours or resulting in death, presumed to be due to cerebral, spinal cord, or retinal tissue injury. Diagnosis was based on clinical symptoms, confirmed by neuroimaging (CT or MRI), or cerebrospinal fluid analysis when necessary. Strokes were categorized into three subtypes:

- Ischemic stroke: caused by prolonged cerebral ischemia leading to infarction. Common etiologies included in situ cerebral artery thrombosis, embolism from the heart or proximal arteries, and global hypoperfusion.
- Hemorrhagic stroke: defined as non-traumatic intracranial bleeding, including intraparenchymal, intraventricular, or subarachnoid hemorrhage.
- Undetermined stroke: cases lacking sufficient clinical or imaging data to distinguish between ischemic and hemorrhagic subtypes.

# Supplementary method 7. Sample size estimation

Sample size was estimated using two approaches: comparison of proportions with unequal group sizes and the log-rank method based on hazard ratio (HR). Both methods were used to ensure sufficient power to detect a clinically meaningful difference in the primary endpoint of 1-year major adverse cardiac events (MACE) between patients with moderate-to-severe versus none/mild CAC.

***Proportion-based method***

Sample size for comparing two independent proportions with unequal group sizes was calculated using the following formula:

$$p=\frac{p_{A}+rp_{B}}{1+r}$$

$$n_{A}\geq\frac{Z_{1-\frac{\propto}{2}}\sqrt{\left( r+1 \right)p(1-p)}+Z_{1-\beta} \sqrt{rp_{A}\left( 1-p_{A} \right)+p_{B}\left( 1-p_{B} \right)}}{r{(p_{B}-p_{A})}^{2}}$$

$n_{B}=n_{A}\times r$ *and* $N_{Total}\geq n_{A}+n_{B}$

Where:

- $\alpha=$ 0.05 (two-sided), $Z_{\left( 1-\frac{\alpha}{2} \right)}=$ 1.96.
- $\beta=$ 0.20, corresponding to 80% power, $Z_{\left( 1-\beta\right)}=$ 0.84.
- $r$: ratio of group sizes.
- $p_{A}$, $p_{B}$: expected event rates in none/mild and moderate/severe CAC groups, respectively.

Based on Copeland-Halperin et al., the estimated 1-year MACE rate was 8.3% in the none/mild CAC group and 16.0% in the moderate/severe CAC group, with a group size ratio of $r=$0.22.^1^ Thus, the required sample sizes were:

- $n_{A}\geq$718, $n_{B}\geq$ 158.
- Minimum total sample size: ≥ 876 patients.

***Hazard ratio–based method***

$$n_{events}\geq\frac{{2\left( Z_{1-\frac{\alpha}{2}}+Z_{1-\beta} \right)}^{2}}{\left( {log}_{e}HR \right)^{2}}; n_{Total}\geq\frac{n_{events}}{Prev}$$

Where:

- $\alpha=$ 0.05 (two-sided), $Z_{\left( 1-\frac{\alpha}{2} \right)}=$ 1.96.
- $\beta=$ 0.20, corresponding to 80% power, $Z_{\left( 1-\beta\right)}=$ 0.84.
- $\mathrm{HR}$ = 1.4: assumed hazard ratio between groups.
- $\mathrm{Prev}$ = 0.183: proportion of patients with moderate-to-severe CAC, based on Zhang et al.^2^

Using this method, the minimum number of events required was 133, corresponding to a total minimum sample size of 727 patients.

***Final sample size***

To ensure sufficient statistical power and account for potential exclusions or follow-up losses, a total of 914 patients were prospectively enrolled in the study.

# Supplementary method 8. STROBE checklist for observational cohort studies

|  | Item No | Recommendation |
| --- | --- | --- |
| **Title and abstract** | 1 | (*a*) Indicate the study’s design with a commonly used term in the title or the abstract ✓ |
|  |  | (*b*) Provide in the abstract an informative and balanced summary of what was done and what was found ✓ |
| Introduction | | |
| Background/rationale | 2 | Explain the scientific background and rationale for the investigation being reported ✓ |
| Objectives | 3 | State specific objectives, including any prespecified hypotheses ✓ |
| Methods | | |
| Study design | 4 | Present key elements of study design early in the paper ✓ |
| Setting | 5 | Describe the setting, locations, and relevant dates, including periods of recruitment, exposure, follow-up, and data collection ✓ |
| Participants | 6 | (*a*) Give the eligibility criteria, and the sources and methods of selection of participants. Describe methods of follow-up ✓ |
|  |  | (*b*) For matched studies, give matching criteria and number of exposed and unexposed ✓ |
| Variables | 7 | Clearly define all outcomes, exposures, predictors, potential confounders, and effect modifiers. Give diagnostic criteria, if applicable ✓ |
| Data sources/ measurement | 8* | For each variable of interest, give sources of data and details of methods of assessment (measurement). Describe comparability of assessment methods if there is more than one group ✓ |
| Bias | 9 | Describe any efforts to address potential sources of bias ✓ |
| Study size | 10 | Explain how the study size was arrived at ✓ |
| Quantitative variables | 11 | Explain how quantitative variables were handled in the analyses. If applicable, describe which groupings were chosen and why ✓ |
| Statistical methods | 12 | (*a*) Describe all statistical methods, including those used to control for confounding ✓ |
|  |  | (*b*) Describe any methods used to examine subgroups and interactions ✓ |
|  |  | (*c*) Explain how missing data were addressed |
|  |  | (*d*) If applicable, explain how loss to follow-up was addressed ✓ |
|  |  | (*e*) Describe any sensitivity analyses ✓ |
| Results | | |
| Participants | 13* | (a) Report numbers of individuals at each stage of study—eg numbers potentially eligible, examined for eligibility, confirmed eligible, included in the study, completing follow-up, and analysed ✓ |
|  |  | (b) Give reasons for non-participation at each stage ✓ |
|  |  | (c) Consider use of a flow diagram ✓ |
| Descriptive data | 14* | (a) Give characteristics of study participants (eg demographic, clinical, social) and information on exposures and potential confounders ✓ |
|  |  | (b) Indicate number of participants with missing data for each variable of interest ✓ |
|  |  | (c) Summarise follow-up time (eg, average and total amount) ✓ |
| Outcome data | 15* | Report numbers of outcome events or summary measures over time ✓ |
| Main results | 16 | (*a*) Give unadjusted estimates and, if applicable, confounder-adjusted estimates and their precision (eg, 95% confidence interval). Make clear which confounders were adjusted for and why they were included ✓ |
|  |  | (*b*) Report category boundaries when continuous variables were categorized ✓ |
|  |  | (*c*) If relevant, consider translating estimates of relative risk into absolute risk for a meaningful time period ✓ |
| Other analyses | 17 | Report other analyses done—eg analyses of subgroups and interactions, and sensitivity analyses ✓ |
| Discussion | | |
| Key results | 18 | Summarise key results with reference to study objectives ✓ |
| Limitations | 19 | Discuss limitations of the study, taking into account sources of potential bias or imprecision. Discuss both direction and magnitude of any potential bias ✓ |
| Interpretation | 20 | Give a cautious overall interpretation of results considering objectives, limitations, multiplicity of analyses, results from similar studies, and other relevant evidence ✓ |
| Generalisability | 21 | Discuss the generalisability (external validity) of the study results ✓ |
| Other information | | |
| Funding | 22 | Give the source of funding and the role of the funders for the present study and, if applicable, for the original study on which the present article is based ✓ |

*Give information separately for exposed and unexposed groups.

*Give information separately for exposed and unexposed groups.

# Supplementary appendix 2. Additional results

# Supplementary result 1. Intra- and Inter-Observer Reproducibility of IVUS-Based Calcification Assessment

Intra- and inter-observer reproducibility of IVUS-based coronary calcification assessment was evaluated in a randomly selected subset of 60 lesions, including 30 lesions with none or mild calcification and 30 with moderate-to-severe calcification, selected by stratified random sampling. IVUS reassessments were independently performed by the primary operator and a second experienced interventional cardiologist, both fully blinded to clinical, angiographic, prior IVUS, and outcome data.

For categorical assessment, agreement was evaluated using weighted Cohen’s kappa for four-level calcification grading and Cohen’s kappa for binary grouping (none–mild vs. moderate–severe). Intra-observer agreement was excellent (weighted κ = 0.841; binary κ = 0.933; overall agreement 96.7%), with similarly high inter-observer agreement (weighted κ = 0.818; binary κ = 0.933). Disagreements were limited to adjacent grading categories.

For quantitative measurements, reproducibility of maximal calcium arc and calcium length was assessed using intraclass correlation coefficients (ICC). Intra-observer reproducibility was excellent for maximal calcium arc (ICC = 0.971) and good for calcium length (ICC = 0.768). Inter-observer reproducibility was likewise high (ICC = 0.920 and 0.880, respectively).

Detailed results are provided in Supplementary table 1 and 2.

# Supplementary result 2. Sensitivity and interaction analyses addressing residual confounding by peripheral artery disease and renal replacement therapy

Because peripheral artery disease and renal replacement therapy remained more prevalent in the moderate-to-severe coronary artery calcification group after propensity score matching, additional sensitivity and interaction analyses were performed.

In the PSM cohort, further adjustment for peripheral artery disease and renal replacement therapy did not alter the primary findings, and moderate-to-severe coronary artery calcification was not independently associated with 1-year major adverse cardiac events (HR 1.072, 95% CI 0.565–2.033; p = 0.832), whereas renal replacement therapy remained a strong predictor of adverse outcomes (HR 11.678, 95% CI 5.495–24.819; p < 0.001).

Formal interaction analyses showed no significant effect modification by peripheral artery disease (CAC × peripheral artery disease: HR 2.106, 95% CI 0.235–18.871; p = 0.506) or by renal replacement therapy (CAC × renal replacement therapy: HR 1.093, 95% CI 0.125–9.573; p = 0.936).

In a sensitivity analysis excluding patients receiving renal replacement therapy, moderate-to-severe coronary artery calcification remained not significantly associated with 1-year major adverse cardiac events (HR 1.122, 95% CI 0.578–2.187; p = 0.733). The results of these analyses are summarized in Supplementary table 3.

# Supplementary result 3. Multivariable Cox regression analysis of factors associated with 1-year MACE

Results from exploratory multivariable analyses in the unmatched cohort are provided in Supplementary table 6. Three hierarchical Cox regression models were constructed to evaluate the independent association between moderate-to-severe coronary calcification and 1-year MACE.

Model 1, which adjusted for age, sex, and comorbidities, showed that age (HR 1.032; 95% CI: 1.004–1.061; P = 0.024), heart failure (HR 1.900; 95% CI: 1.005–3.593; P = 0.048), and chronic kidney disease (HR 2.368; 95% CI: 1.266–4.431; P = 0.007) were significantly associated with increased risk of MACE. Coronary calcification was not independently associated with outcome in this model (P = 0.167).

In Model 2, which included admission diagnosis (acute coronary syndrome or acute heart failure), acute heart failure remained a strong predictor of MACE (HR 4.317; 95% CI: 2.274–7.527; P < 0.001), and both age and chronic kidney disease remained significant. Coronary calcification did not reach statistical significance (P = 0.248).

Model 3 further adjusted for angiographic lesion characteristics, including SYNTAX score, multivessel disease, and left main involvement. In this fully adjusted model, acute coronary syndrome (HR 3.120; 95% CI: 1.726–5.642; P < 0.001), age (P = 0.043), and chronic kidney disease (P = 0.034) remained independently associated with 1-year MACE. Coronary calcification was not an independent predictor after full adjustment (P = 0.556).

These findings suggest that although coronary calcification is associated with adverse outcomes in unadjusted analyses, its prognostic impact is attenuated after controlling for key clinical and angiographic confounders.

# Supplementary result 4. Impact of IVUS-defined stent optimization stratified by CAC severity

Supplementary table 7 and Supplementary figure 1 present the impact of IVUS-defined stent optimization on 1-year clinical outcomes stratified by CAC severity.

In the propensity score–matched cohort, among patients with moderate-to-severe CAC, achievement of IVUS-defined optimal stent implantation was associated with a 1-year MACE rate comparable to that observed in patients with none or mild CAC who also achieved optimal IVUS results (HR = 1.43; p = 0.340).

In contrast, patients with moderate-to-severe CAC who failed to achieve IVUS-defined stent optimization experienced a substantially higher risk of 1-year MACE. Specifically, the risk was approximately 2.9-fold higher compared with patients with none or mild CAC who achieved optimal IVUS results, and approximately 1.5-fold higher compared with patients with moderate-to-severe CAC who achieved optimal stent deployment.

# Supplementary result 5. Impact of ACS Status on the Association Between CAC Severity and 1-Year MACE

To assess whether ACS status influenced the association between moderate-to-severe CAC and 1-year outcomes, we performed subgroup, interaction, and sensitivity analyses.

In subgroup analyses, the association between CAC severity and 1-year MACE remained non-significant in both MI (STEMI/NSTEMI) and non-MI (UA/CCS) groups. In the interaction model, the CAC×MI term was also non-significant, indicating that MI presentation did not modify the effect of CAC severity on clinical outcomes.

A multivariable Cox model restricted to MI patients yielded similar results, with moderate-to-severe CAC showing no independent association with 1-year MACE.

Together, these findings demonstrate that ACS status does not alter the relationship between CAC severity and adverse outcomes. Summary results are presented in Supplementary table 8.

# Supplementary tables

# Supplementary table 1. Intra- and inter-observer reproducibility of categorical IVUS-based coronary calcification grading (n = 60)

|  | **Statistical method** | **Agreement metric (95% CI)** | **Overall agreement (%)** |
| --- | --- | --- | --- |
| Intra-observer | | | |
| CAC grading (4 levels: none, mild, moderate, severe) | Weighted Cohen’s kappa | $\kappa=$ 0.841  (0.731 – 0.951) | 88.3 |
| CAC grouping (none–mild vs. moderate–severe) | Cohen’s kappa | $\kappa=$ 0.933  (0.843 – 1.000) | 96.7 |
| Calcified nodule | Cohen’s kappa | $\kappa=$ 0.775  (0.591 – 0.959) | 91.7 |
| Inter-observer | | | |
| CAC grading (4 levels: none, mild, moderate, severe) | Weighted Cohen’s kappa | $\kappa=$ 0.818  (0.702 – 0.934) | 86.7 |
| CAC grouping (none–mild vs. moderate–severe) | Cohen’s kappa | $\kappa=$ 0.933  (0.843 – 1.000) | 96.7 |
| Calcified nodule | Cohen’s kappa | $\kappa=$ 0.762  (0.566 – 0.958) | 91.7 |

Weighted Cohen’s kappa was used for ordinal categorical variables; Cohen’s kappa was used for binary variables.

# Supplementary table 2. Intra- and inter-observer reproducibility of quantitative IVUS-based coronary calcification measurements (n = 60)

|  | **ICC (95% CI)**  **(single)** | **ICC (95% CI)**  **(average)** |
| --- | --- | --- |
| Intra-observer | | |
| Maximal calcium arc (degrees) | 0.971 (0.953 – 0.983) | 0.986 (0.976 – 0.991) |
| Calcium length (mm) | 0.768 (0.638 – 0.855) | 0.869 (0.779 – 0.922) |
| Inter-observer | | |
| Maximal calcium arc (degrees) | 0.920 (0.870 – 0.952) | 0.958 (0.930 – 0.975) |
| Calcium length (mm) | 0.880 (0.807 – 0.926) | 0.936 (0.893 – 0.962) |

# Intraclass correlation coefficients (ICCs) were calculated using two-way random-effects models with an absolute agreement definition. For intra-observer analysis, measurements were repeated by the same observer; for inter-observer analysis, measurements were performed independently by two observers. Average-measure ICCs represent the mean of two measurements.

# Supplementary table 3. Sensitivity and interaction analyses evaluating residual confounding and effect modification by peripheral artery disease and renal replacement therapy

|  | **HR** | **95% CI** | **p value** |
| --- | --- | --- | --- |
| Adjusted Cox analysis | | | |
| Renal replacement therapy | 11.678 | 5.495 – 24.819 | <0.001 |
| Peripheral artery disease | 1.644 | 0.748 – 3.614 | 0.216 |
| Moderate-to-severe CAC | 1.072 | 0.565 – 2.033 | 0.832 |
| Interaction analysis (CAC x Renal replacement therapy) | | | |
| Moderate-to-severe CAC × Renal replacement therapy^*^ | 1.093 | 0.125 – 9.573 | 0.936 |
| Moderate-to-severe CAC (main effect) | 1.125 | 0.580 – 2.182 | 0.728 |
| Renal replacement therapy (main effect) | 11.284 | 1.498 – 84.985 | 0.019 |
| Interaction analysis (CAC x Peripheral artery disease) | | | |
| Moderate-to-severe CAC x Peripheral artery disease^*^ | 2.106 | 0.235 – 18.871 | 0.506 |
| Moderate-to-severe CAC (main effect) | 1.322 | 0.693 – 2.524 | 0.397 |
| Peripheral artery disease (main effect) | 1.057 | 0.141 – 7.943 | 0.957 |
| Sensitivity analysis excluding Renal replacement therapy | | | |
| Moderate-to-severe CAC | 1.122 | 0.578 – 2.187 | 0.733 |

All analyses were performed in the propensity score–matched cohort unless otherwise specified.

*Hazard ratios represent interaction terms.

CAC: coronary artery calcification; HR: hazard ratio; CI: confidence interval.

# Supplementary table 4. Laboratory characteristics

|  | **Before PSM** | | | **After PSM** | | |
| --- | --- | --- | --- | --- | --- | --- |
|  | No/mild calcification  (n=576) | Moderate/severe calcification  (n=338) | P value | No/mild calcification  (n=288) | Moderate/severe calcification  (n=288) | P value |
| Total cholesterol (mg/dL) | 194.0 (163.0 – 227.0) | 174.0 (137.8 – 259.6) | 0.123 | 192.0 (157.0 – 218.5) | 174.0 (139.0 – 204.0) | < 0.001 |
| LDL cholesterol (mg/dL) | 127.0 (102.0 – 151.0) | 113.5 (83.0 – 139.0) | 0.215 | 124.0 (99.0 – 148.0) | 114.0 (90.0 – 137.0) | 0.001 |
| HDL cholesterol (mg/dL) | 41.0 (36.0 – 47.0) | 40.0 (33.0 – 47.0) | 0.786 | 42.0 (36.0 – 48.0) | 39.5 (33.0 – 46.0) | 0.232 |
| Triglycerides (mg/dL) | 160.0 (111.0 – 247.0) | 142.2 (100.8 – 199.0) | 0.098 | 158.0 (112.5 – 237.5) | 145.5 (107.0 – 206.0) | 0.001 |
| Creatinine (mg/dL) | 0.95 (0.83 – 1.11) | 1.0 (0.84 – 1.28) | 0.735 | 1.0 (0.8 – 1.2) | 1.0 (0.8 – 1.3) | 0.907 |
| eGFR (mL/min/1.73 m²) | 69.6$\pm$24.8 | 53.4$\pm$22.3 | 0.013 | 57.7$\pm$21.5 | 56.5$\pm$22.4 | 0.526 |
| eGFR < 30 (mL/min/1.73 m²) | 28 (4.9) | 43 (12.7) | < 0.001 | 26 (9.0) | 32 (11.1) | 0.406 |
| CRP (mg/L) | 6.1 (2.6 – 15.7) | 9.4 (3.9 – 29.0) | 0.482 | 6.5 (3.1 – 6.5) | 9.1 (4.1 – 26.7) | 0.240 |
| CKMB (U/L) | 39.0 (18.0 – 111.5) | 26.0 (16.0 – 69.0) | 0.221 | 38.0 (18.0 – 87.5) | 26.0 (16.0 – 70.0) | 0.614 |
| hs-Troponin T (ng/L) | 11153.0 (232.5 – 3913.0) | 742.0 (126.0 – 3220.0) | 0.362 | 1128.0 (270.0 – 3610.5) | 587.0 (112.0 – 3185.0) | 0.742 |
| NT-proBNP (ng/L) | 446.0 (110.0 – 1781.0) | 1122.0 (221.0 – 4087.0) | 0.154 | 797.0 (270.5 – 2622.5) | 972.0 (186.0 – 4015.0) | 0.186 |
| Glucose  (mg/dL) | 128.0 (99.0 – 169.5) | 139.0 (103.0 – 191.0) | 0.776 | 135.0 (104.5 – 176.5) | 137.0 (103.0 – 182.0) | 0.512 |
| HbA1c (%) | 6.1 (5.7 – 7.6) | 6.7 (5.9 – 8.1) | 0.898 | 6.4 (5.8 – 7.8) | 6.7 (5.8 – 8.0) | 0.195 |
| Hemoglobin (g/dL) | 138.0$\pm$18.7 | 129.9$\pm$18.0 | 0.618 | 131.9$\pm$17.9 | 130.1$\pm$18.0 | 0.227 |
| LVEF (%) | 55.0 (45.0 – 63.3) | 52.0 (40.0 – 61.0) | 0.209 | 53.0 (43.0 – 60.9) | 52.0 (42.0 – 61.0) | 0.687 |
| LVEF < 40% | 61 (11.2) | 66 (20.5) | < 0.001 | 39 (44.8) | 48 (17.6) | 0.292 |

Values are n (%), mean ± SD, or median (Q1-Q3).

# CKMB, creatine kinase-MB; CRP, C-reactive protein; eGFR, estimated glomerular filtration rate; HbA1c, hemoglobin A1c; HDL, high-density lipoprotein; hs-Troponin T, high-sensitivity troponin T; LDL, low-density lipoprotein; LVEF, left ventricular ejection fraction; NT-proBNP, N-terminal pro–B-type natriuretic peptide; PSM, propensity score matching.

# Supplementary table 5. Univariable Cox regression for 1-year MACE

|  | **HR** | **95% CI** | **P value** |
| --- | --- | --- | --- |
| Baseline Characteristics | | | |
| Age (years) | 1.033 | 1.001 – 1.007 | 0.044 |
| Male | 0.715 | 0.397 – 1.287 | 0.263 |
| Hypertension | 0.892 | 0.398 – 1.998 | 0.782 |
| Prior MI | 0.932 | 0.434 – 2.001 | 0.857 |
| Prior PCI | 0.919 | 0.363 – 2.329 | 0.859 |
| Heart failure | 1.534 | 0.777 – 3.028 | 0.217 |
| Atrial fibrillation | 1.102 | 0.267 – 4.550 | 0.893 |
| Diabetes mellitus | 0.740 | 0.398 – 1.375 | 0.341 |
| Chronic kidney disease | 3.226 | 1.735 – 5.996 | <0.001 |
| Renal replacement therapy | 12.784 | 6.297 – 25.955 | <0.001 |
| Prior Stroke | 1.165 | 0.460 – 2.951 | 0.748 |
| Peripheral artery disease | 2.090 | 0.973 – 4.487 | 0.059 |
| COPD | 1.259 | 0.390 – 4.062 | 0.700 |
| Clinical diagnosis | | | |
| ACS | 1.801 | 0.645 – 5.029 | 0.261 |
| Acute heart failure | 4.510 | 2.449 – 8.307 | <0.001 |
| Laboratory Characteristics | | | |
| Creatinine | 1.366 | 1.254 – 1.487 | <0.001 |
| LVEF | 0.970 | 0.951 – 0.990 | 0.004 |
| LVEF < 40% | 1.992 | 0.998 – 3.974 | 0.051 |
| Coronary Lesion Characteristics | | | |
| Multi-vessel disease | 2.728 | 0.845 – 8.800 | 0.093 |
| LM disease | 1.041 | 0.710 – 1.525 | 0.838 |
| SYNTAX score | 1.024 | 0.993 – 1.055 | 0.130 |
| Coronary calcification | 1.538 | 0.847 – 2.792 | 0.157 |
| Optimal stent implantation | 0.527 | 0.286 – 0.970 | 0.040 |

Values are n (%), mean ± SD, or median (Q1-Q3).

ACS: acute coronary syndrome; CI: confidence interval; COPD: chronic obstructive pulmonary disease; HR: hazard ratio; LM: left main; LVEF: left ventricular ejection fraction; mi: myocardial infarction; PCI: percutaneous coronary intervention.

# Supplementary table 6. Multivariable Cox regression analysis of factors associated with 1-year MACE

|  | **HR** | **95% CI** | **P value** |
| --- | --- | --- | --- |
| Model 1: Adjusted for age, sex, and comorbidities | | | |
| Age (per year) | 1.032 | 1.004 – 1.061 | 0.024 |
| Male | 0.885 | 0.503 – 1.560 | 0.673 |
| Hypertension | 1.113 | 0.504 – 2.458 | 0.791 |
| Prior MI | 0.760 | 0.364 – 1.584 | 0.464 |
| Heart failure | 1.900 | 1.005 – 3.593 | 0.048 |
| Diabetes mellitus | 0.656 | 0.370 – 1.165 | 0.150 |
| Chronic kidney disease | 2.368 | 1.266 – 4.431 | 0.007 |
| Coronary calcification | 1.509 | 0.842 – 2.706 | 0.167 |
| Model 2: Model 1 + Admission status | | | |
| Age (per year) | 1.028 | 1.002 – 1.054 | 0.037 |
| Diabetes mellitus | 0.573 | 0.322 – 1.021 | 0.059 |
| Chronic kidney disease | 2.119 | 1.137 – 3.948 | 0.018 |
| Heart failure | 1.638 | 0.888 – 3.022 | 0.114 |
| Acute coronary syndrome | 1.084 | 0.444 – 2.644 | 0.859 |
| Acute heart failure | 4.317 | 2.274 – 7.527 | < 0.001 |
| Coronary calcification | 1.415 | 0.785 – 2.552 | 0.248 |
| Model 3: Model 2 + Angiographic lesion characteristics | | | |
| Age (per year) | 1.027 | 1.001 – 1.054 | 0.043 |
| Chronic kidney disease | 1.943 | 1.051 – 3.591 | 0.034 |
| Heart failure | 1.451 | 0.775 – 2.715 | 0.245 |
| Acute coronary syndrome | 3.120 | 1.726 – 5.642 | < 0.001 |
| SYNTAX score | 1.010 | 0.975 – 1.047 | 0.582 |
| Multivessel disease | 1.830 | 0.695 – 4.816 | 0.221 |
| Left main disease | 0.996 | 0.489 – 2.027 | 0.991 |
| Coronary calcification | 1.204 | 0.649 – 2.234 | 0.556 |

CI: confidence interval; HR: hazard ratio; MI: myocardial infarction

# Supplementary table 7. Association between IVUS-defined stent optimization and 1-year MACE stratified by coronary artery calcification severity

|  | **MACE, n (%)** | **HR** | **95% CI** | **P value** |
| --- | --- | --- | --- | --- |
| Before PSM (N = 910) | | | | |
| None/mild CAC + IVUS optimal (n = 459) | 17 (3.7) | Reference | | |
| None/mild CAC + IVUS suboptimal (n = 116) | 7 (6.0) | 1,652 | 0.685 – 3.983 | 0.264 |
| Moderate-to-severe CAC + IVUS optimal (n = 229) | 17 (7.4) | 2,061 | 1.052 – 4.037 | 0.035 |
| Moderate-to-severe CAC + IVUS suboptimal (n = 106) | 14 (13.2) | 3,787 | 1.867 – 7.683 | < 0.001 |
| After PSM (N = 576) | | | | |
| None/mild CAC + IVUS optimal (n = 227) | 13 (5.7) | Reference | | |
| None/mild CAC + IVUS suboptimal (n = 61) | 5 (8.2) | 1.466 | 0.523 – 4,112 | 0.467 |
| Moderate-to-severe CAC + IVUS optimal (n = 88) | 16 (8.0) | 1.428 | 0.687 – 2.968 | 0.340 |
| Moderate-to-severe CAC + IVUS suboptimal (n = 200) | 11 (24.4) | 2.923 | 1.027 – 5.118 | 0.043 |

CAC: coronary artery calcification; CI: confidence interval; HR: hazard ratio; IVUS: intravascular ultrasound; MACE: major adverse cardiac events; PSM: propensity score matching.

|  | **HR** | **95% CI** | **p value** |
| --- | --- | --- | --- |
| Subgroup analysis: Association between moderate-to-severe CAC and 1-Year MACE in MI and non-MI patients | | | |
| MI (STEMI + NSTEMI) | 1.524 | 0.790 – 2.937 | 0.208 |
| Non-MI (UA + CCS) | 1.672 | 0.400 – 6.996 | 0.482 |
| Interaction analysis between moderate-to-severe CAC and MI presentation | | | |
| Moderate-to-severe CAC × MI presentation^*^ | 0.906 | 0.188 – 4.376 | 0.902 |
| Moderate-to-severe CAC (main effect) | 1.681 | 0.402 – 7.036 | 0.477 |
| MI presentation (main effect) | 3.935 | 1.139 – 13.593 | 0.030 |
| Sensitivity analysis: multivariable Cox model restricted to MI patients | | | |
| Moderate-to-severe CAC | 1.247 | 0.618 – 2.514 | 0.538 |
| Age (per year) | 1.029 | 0.992 – 1.068 | 0.130 |
| Chronic kidney disease | 2.354 | 1.133 – 4.888 | 0.022 |
| Peripheral artery disease | 2.082 | 0.863 – 5.021 | 0.103 |
| Acute heart failure | 2.299 | 1.167 – 4.526 | 0.016 |
| Multi-vessel disease | 1.787 | 0.534 – 5.978 | 0.346 |
| Left main disease | 0.546 | 0.205 – 1.453 | 0.226 |
| Optimal stent implantation | 0.695 | 0.349 – 1.384 | 0.301 |

# Supplementary table 8. Impact of ACS status on the association between moderate-to-severe CAC and 1-Year MACE

All analyses were performed in the propensity score–matched cohort unless otherwise specified.

*Hazard ratios represent interaction terms.

CAC: coronary artery calcification; HR: hazard ratio; CI: confidence interval; MI: myocardial infarction

# Supplementary Figures


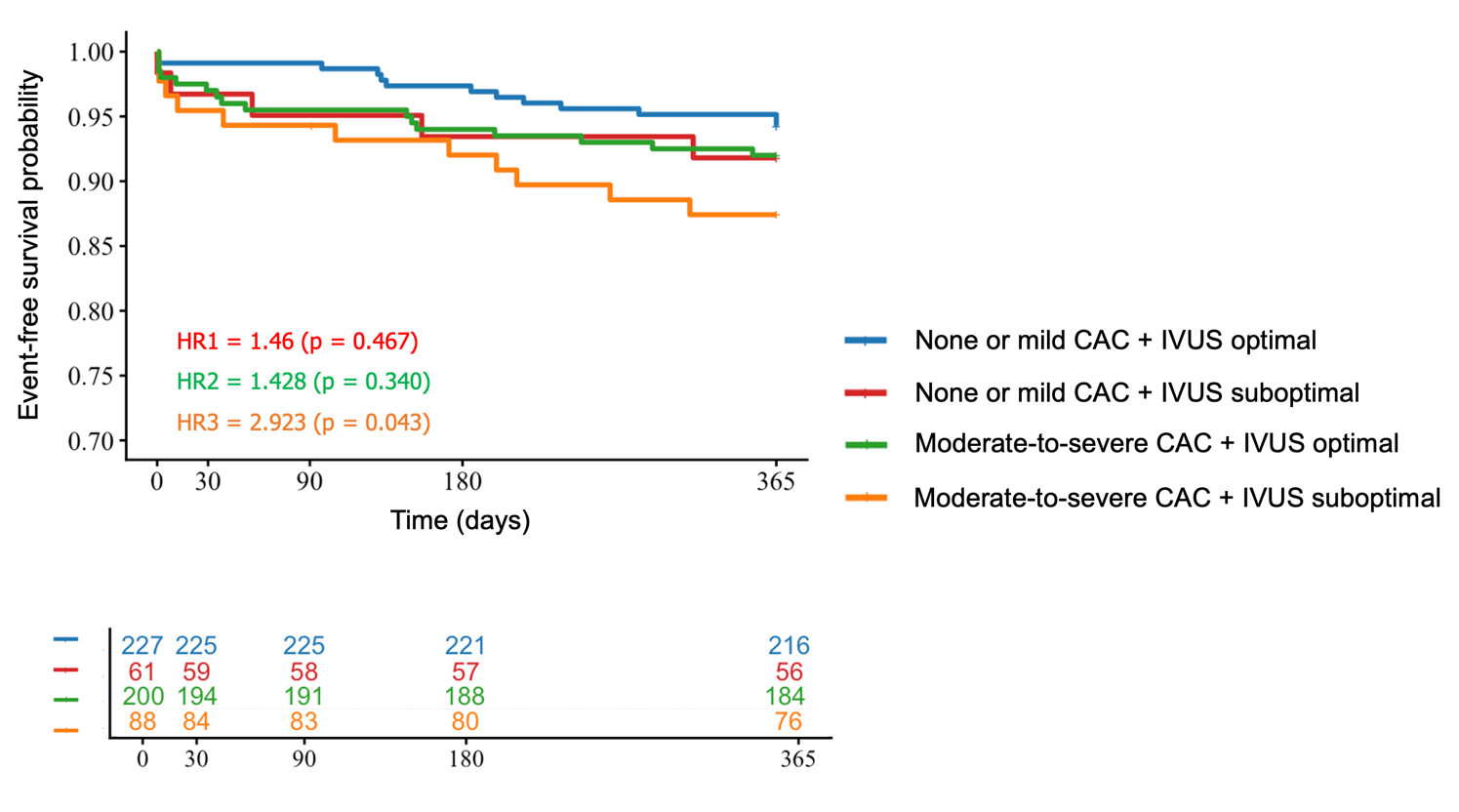


**Supplementary Figure 1.** Kaplan–Meier curves for 1-year MACE stratified by coronary artery calcification severity and IVUS-defined stent optimization after propensity score matching.

**References**

1. Räber L, Mintz GS, Koskinas KC, Johnson TW, Holm NR, Onuma Y, Radu MD, Joner M, Yu B, Jia H, Meneveau N, de la Torre Hernandez JM, Escaned J, Hill J, Prati F, Colombo A, di Mario C, Regar E, Capodanno D, Wijns W, Byrne RA, Guagliumi G, Group ESD. Clinical use of intracoronary imaging. Part 1: guidance and optimization of coronary interventions. An expert consensus document of the European Association of Percutaneous Cardiovascular Interventions. European Heart Journal. 2018;39(35):3281-3300.

2. Saito Y, Kobayashi Y, Fujii K, Sonoda S, Tsujita K, Hibi K, Morino Y, Okura H, Ikari Y, Kozuma K. CVIT 2025 clinical expert consensus document on intravascular ultrasound. Cardiovascular Intervention and Therapeutics. 2025;40(2):211-225.

3. Bangalore S, Maehara A. Intravascular ultrasound optimization criteria for left main coronary artery stenting: in pursuit of the magic numbers! : Lippincott Williams & Wilkins Hagerstown, MD; 2024:e013691.
